# HIV-1 Vpr Induces Widespread Transcriptomic Changes in CD4^+^ T Cells Early Postinfection

**DOI:** 10.1128/mBio.01369-21

**Published:** 2021-06-22

**Authors:** Hélène Bauby, Christopher C. Ward, Rupert Hugh-White, Chad M. Swanson, Reiner Schulz, Caroline Goujon, Michael H. Malim

**Affiliations:** a Department of Infectious Diseases, School of Immunology and Microbial Sciences, King’s College London, London, United Kingdom; Columbia University/HHMI

**Keywords:** HIV, transcriptomics, Vpr, virus-host interactions

## Abstract

The interactions between a virus and its host are complex but can be broadly categorized as either viral manipulation of cellular functions or cellular responses to infection. These processes begin at the earliest point of contact between virus and cell and frequently result in changes to cellular gene expression, making genome-wide transcriptomics a useful tool to study them. Several previous studies have used transcriptomics to evaluate the cellular responses to human immunodeficiency virus type 1 (HIV-1) infection; however, none have examined events in primary CD4^+^ T cells during the first 24 h of infection. Here, we analyzed CD4^+^ T cells at 4.5, 8, 12, 24, and 48 h following infection. We describe global changes to host gene expression commencing at 4.5 h postinfection and evolving over the ensuing time points. We identify upregulation of genes related to innate immunity, cytokine production, and apoptosis and downregulation of those involved in transcription and translation. We further demonstrate that the viral accessory protein Vpr is necessary for almost all gene expression changes seen at 12 h postinfection and the majority of those seen at 48 h. Identifying this new role for Vpr not only provides fresh perspective on its possible function but also adds further insight into the interplay between HIV-1 and its host at the cellular level.

## INTRODUCTION

As obligate intracellular parasites, viruses require many elements of cellular machinery to successfully complete their life cycles, often referred to as dependency factors. Beyond this, to replicate efficiently, it is often necessary for viruses to actively modify host cell processes from the earliest point of contact with the cell. Illustrative examples of such viral manipulation of early events include vaccinia virus initiation of macropinocytosis by mimicry of apoptotic bodies, use of multiple cellular kinases by simian virus 40 (SV40) to promote entry through caveolar endocytosis and transport to the smooth endoplasmic reticulum, and repression of cellular transcription by the matrix protein of vesicular stomatitis virus (1–5). Conversely, host cells have evolved myriad mechanisms to impede viral infection at the earliest possible opportunity and limit subsequent damage; the antiviral protein effectors of inhibition are often referred to as restriction factors (6). The wide and varied signaling cascades that ensue from these interactions inevitably change cellular transcription and RNA expression, making transcriptomics an important approach for acquiring a broad and unbiased overview of host-pathogen interactions.

A number of prior studies have assessed transcriptomic changes following human immunodeficiency virus type 1 (HIV-1) infection in different contexts. Studies can be grouped into those examining gene expression *in vivo* in infected individuals and those examining RNA levels following infection of cells *in vitro*. The *in vivo* studies of humans or following simian immunodeficiency virus infection of nonhuman primates have shown an upregulation of genes associated with innate immunity. Many of these studies have also noted differential expression of genes related to the cell cycle but have differed in their conclusions as to the consequences of these changes (7–9).

Transcriptomic studies of cells infected *in vitro* with HIV-1 can be further divided into those studies performed using immortalized cell lines and those performed on primary CD4^+^ T cells *ex vivo*. There are many studies of the former genre, and these have been reviewed elsewhere (10, 11); in sum, no unifying picture of genome-wide changes in RNA expression has emerged, perhaps due to the use of different cell lines, viral isolates, and experimental workflows. To our knowledge, three studies using primary CD4^+^ T cells and full-length infectious virus have been performed. The first concluded that effector memory T cells and cells with high levels of active AP-1 (a transcription factor) were more permissive for infection (12). The second examined changes to transcription at 48 h postinfection and identified increases in the expression of genes related to innate immunity (13). Significant differential expression of genes related to apoptosis and cytokine production was also noted. A third, more recent, study sought to assess the impact of Vpu on cellular gene expression 72 h postinfection; however, following infection with wild-type virus, this study demonstrated upregulation of genes associated with antiviral immunity and downregulation of genes in ontology categories, including the spliceosome, RNA transport, and ribosome biogenesis (14).

Given the potential of transcriptomics to identify dependency and restriction factors, we employed this approach to investigate the very earliest events during infection. While one previous study documented transcriptional changes in the first 24 h of HIV-1 infection in SupT1 cells (15), none have investigated primary T cells. We therefore infected primary CD4^+^ T cells with HIV-1 and assessed genome-wide RNA expression at 4.5, 8, 12, 24, and 48 h following infection. Specific changes were evident by 4.5 h, and the identities and functions of affected genes varied over the subsequent time points. Further infections using primary memory CD4^+^ T cells and different HIV-1 strains, including transmitted founder viruses, confirmed these observations. Finally, we sought to identify the viral factors responsible for initial transcriptomic reprogramming and, for the first time, defined the accessory protein Vpr as necessary for almost all cellular gene expression changes occurring within the first 12 h of HIV-1 infection.

## RESULTS

### HIV-1 induces rapid genome-wide transcriptome changes following infection of primary CD4^+^ T cells.

**FIG 1:**
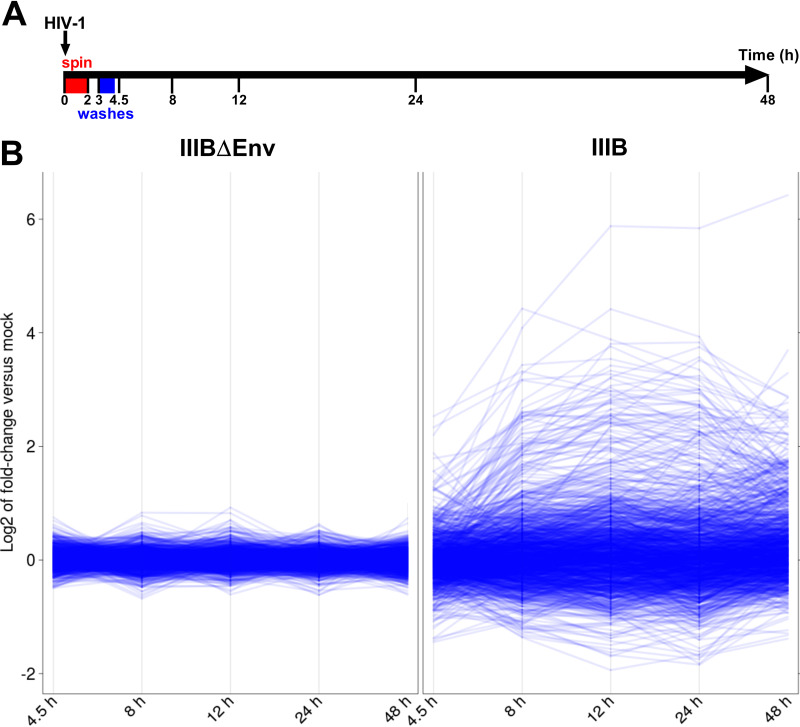
HIV-1 induces genome-wide transcriptome changes in primary CD4^+^ T cells following infection. CD4^+^ T cells were isolated from healthy donors and stimulated for 40 h with soluble anti-CD3 and anti-CD28 antibodies before spin infection with IIIB, IIIBΔEnv, or no virus. (A and B) RNA was extracted at 4.5, 8, 12, 24, or 48 h postinfection (A), and gene expression was assessed using Illumina’s BeadArray HT12v4 (B). (B) Line plots that illustrate the qualitative difference in the degrees of observed gene expression changes between IIIBΔEnv- (left) and IIIB-infected (right) cells relative to mock-infected cells. Each line corresponds to the gene expression changes detected by a particular microarray probe at each of the five time points. The set of probes shown on the left and right is the same (*n* = 1,303) and corresponds to the 5% of microarray probes that detected the greatest gene expression changes overall. Specifically, all probes were ranked according to the amount of gene expression change (F statistic) detected across the 10 pairwise comparisons (two by five time points) between mock-infected and either IIIBΔEnv- (left) or IIIB-infected (right) cells. Therefore, probe selection was independent of experimental condition. Nevertheless, the selected 5% of probes detected gene expression changes greater than 2-fold in IIIB- but not in IIIBΔEnv-infected cells. Note that, while overall differential expression for a probe may have been high, differential expression at an individual time point may still be zero.

There has been limited analysis of the primary CD4^+^ T-cell transcriptional response to HIV-1 infection *in vitro*, and prior work has focused on gene expression changes occurring between 24 and 72 h postinfection (12–14). Other studies have used viral antigen (e.g., gp120) or T-cell lines to examine transcriptional responses in the first 24 h postchallenge (15–17). We sought to build upon these observations by examining responses in primary CD4^+^ T cells using replication-competent virus at multiple early time points across multiple donors. Primary CD4^+^ T cells were isolated from the blood of healthy volunteers and activated for 40 h before challenge with either no virus (mock), the wild-type CXCR4 (X4)-tropic strain HIV-1_IIIB_ (herein called IIIB), or an engineered derivative lacking a functional *env* gene, HIV-1_IIIBΔEnv_ (IIIBΔEnv), that is unable to enter cells. Excess input virus was removed by extensive washes with phosphate-buffered saline (PBS) 3 h postinfection, and an aliquot of cells was used to determine the efficiency of infection by intracellular p24^Gag^ staining and flow cytometry. On average, 81% of cells exposed to wild-type IIIB were positive for intracellular p24^Gag^, whereas less than 1% were stained following IIIBΔEnv challenge (see Fig. S2 in the supplemental material). Subsequently, at 4.5, 8, 12, 24, and 48 h postinfection, cells were harvested, total RNA was isolated, and gene expression was determined using Illumina’s BeadArray HT12v4 (Fig. 1A). Of the 47,324 probes on the array (representing 34,697 gene loci), 26,079 detected expression significantly above the background noise level, defined as the global median signal intensity, under at least one of our experimental conditions (false-discovery rate [FDR] < 20%). They represent the gene expression “universe” of our studies.

10.1128/mBio.01369-21.2 FIG S2 Representative flow cytometry plots of assessment of intracellular p24^Gag^. Three hours after infection, aliquots of all samples were fixed, permeabilized, and stained with an anti-p24^Gag^ antibody (KC57-RD1). The percentage of p24^Gag^ positive cells was determined by identifying the proportion of cells showing higher fluorescence in the PE channel (right) than in mock-infected cells. Download FIG S2, TIF file, 0.7 MB. Copyright © 2021 Bauby et al. 2021 Bauby et al. https://creativecommons.org/licenses/by/4.0/ This content is distributed under the terms of the Creative Commons Attribution 4.0 International license.

**FIG 2:**
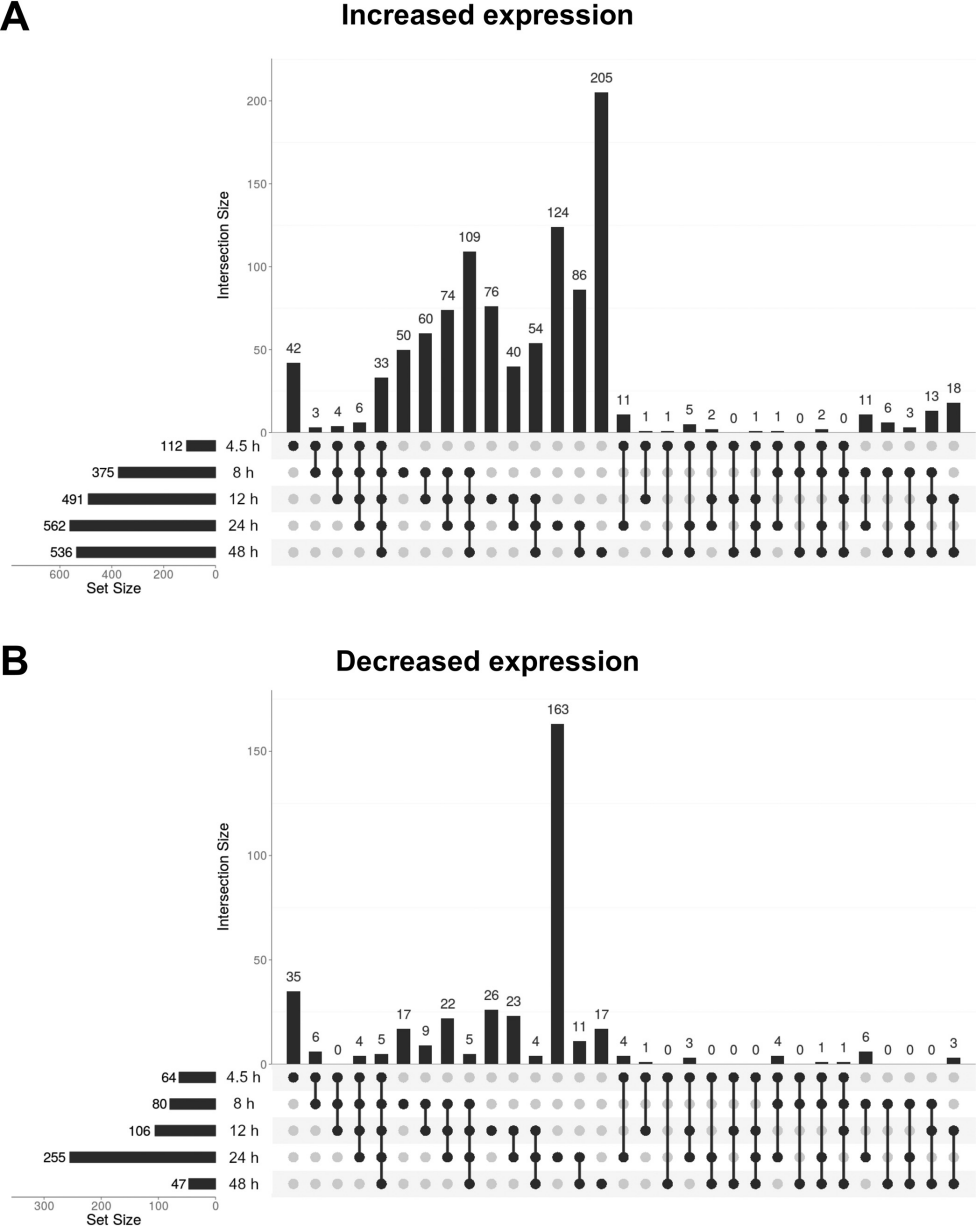
Analysis of probes detecting significantly (FDR < 5%, LFC > 1) increased (A) or decreased (B) expression in IIIB-infected cells relative to controls. Horizonal bars demonstrate the total number of probes detecting a significant change in expression at each time point. Vertical bars illustrate the sizes of all possible mutually exclusive and potentially nonempty subsets of probes that can be generated via the intersection of the five time point-specific sets of probes (*n* = 2^5^ – 1 = 31). By definition, the probes in a particular subset detected significant change in expression at a specific set of time points, indicated below the vertical bar by a column of filled black circles connected by lines, while not detecting significant change at the other time points, indicated by the unconnected light-gray circles in the same column. This is equivalent to a five-way Venn diagram with areas proportional in size to the numbers of probes in each subset. For example, 112 probes in total detected significant upregulation at 4.5 h after infection (first horizontal bar). Of these, 42 probes (1st column) did so exclusively at 4.5 h, while 3, 4, 6, and 33 probes (2nd to 5th columns) did so exclusively at 4.5 and 8 h, 4.5 and 8 and 12 h, 4.5 and 8 and 12 and 24 h, and all time points, respectively.

Across the entire time course, we found the mock infection and IIIBΔEnv experimental conditions to yield equivalent results. Even at an FDR threshold of 80%, at most 2 probes detected large (absolute log_2_ fold change [|LFC|] > 1) expression differences between mock- and IIIBΔEnv-infected cells at any of the time points (Fig. S3). We therefore used both conditions to control for infection-unrelated, time-driven gene expression changes. A total of 5,280 probes detected significant (FDR < 5%) expression changes in cells infected with wild-type virus at some point during the time course relative to the expression levels in these controls (Data Set S1). Of these probes, 1,411 detected large changes (|LFC| > 1), starting with 176 probes at 4.5 h postinfection (Fig. 1B and 2A and B). By 8 h postinfection, this number had increased to 455, reaching a peak of 817 probes at 24 h and decreasing to 583 probes by 48 h.

10.1128/mBio.01369-21.3 FIG S3 Infection with IIIB, but not IIIBΔEnv, causes changes to gene expression compared to that after mock infection. Quantile-quantile plots for comparisons of gene expression between the IIIBΔEnv versus mock (black) and between the IIIB versus IIIBΔEnv and mock (red) experimental conditions, separately for each time point. Each plot states the numbers of microarray probes detecting large gene expression changes (absolute log_2_ fold change |LFC| > 1) with an FDR of <80% for the IIIBΔEnv versus mock comparison and with an FDR of <5% for the IIIB versus IIIBΔEnv plus mock comparison. Download FIG S3, TIF file, 0.2 MB. Copyright © 2021 Bauby et al. 2021 Bauby et al. https://creativecommons.org/licenses/by/4.0/ This content is distributed under the terms of the Creative Commons Attribution 4.0 International license.

10.1128/mBio.01369-21.4 DATA SET S1 Probes detecting significant (FDR < 5%) expression changes in cells infected with wild-type virus relative to expression in control samples at some point during the full-time-course experiments. Download Data Set S1, XLSX file, 1.3 MB. Copyright © 2021 Bauby et al. 2021 Bauby et al. https://creativecommons.org/licenses/by/4.0/ This content is distributed under the terms of the Creative Commons Attribution 4.0 International license.

The identities of the genes affected by infection varied over time, sometimes within relatively short time spans. Considering all intersections between the time point-specific sets of probes detecting either up- or downregulation of gene expression (Fig. 2A and B), we found that, for example, at 4.5 h, most differentially expressed genes (DEGs) fell into two categories: genes either up- or downregulated exclusively at 4.5 h (77 of 176 probes) and upregulated genes that remained so through to the 48-h time point (33 of 176 probes). The four largest intersections also pointed to dynamic changes in the set of DEGs over time; an exceptionally large group of genes (163 probes) were downregulated only at 24 h, accompanied by a large group of upregulated genes (124 probes) that also were specific to the 24-h time point (including a notable upregulation of interferon [IFN]-stimulated genes [ISGs]) (Fig. 2A). Genes upregulated from 8 h onwards gave rise to the largest group of genes (109 probes), which remained upregulated for the rest of the time course; finally, many upregulated DEGs (205 probes) were observed only at 48 h, not earlier. There were small numbers of genes that appeared to be upregulated or downregulated at nonconsecutive time points. While this may be an artifact of the analysis generated by setting a 2-fold cutoff, the possibility that these genes truly have cyclical expression cannot be excluded. It should be noted that the frequency with which genes were observed to be differentially expressed at any combination of nonconsecutive time points was significantly less than that which would be observed by chance (*P* < 1 × 10^−66^). Therefore, even if all of these observations represent true cycle expression, this phenomenon occurs significantly less frequently than differential expression at consecutive time points. In sum, HIV-1 infection and entry into CD4^+^ T cells triggers rapid, dynamic, and genome-wide changes in the host transcriptome; as a general trend, the magnitude of expression changes tended to be greater for upregulated genes than for downregulated genes (Fig. 1B).

### WGCNA.

**FIG 3:**
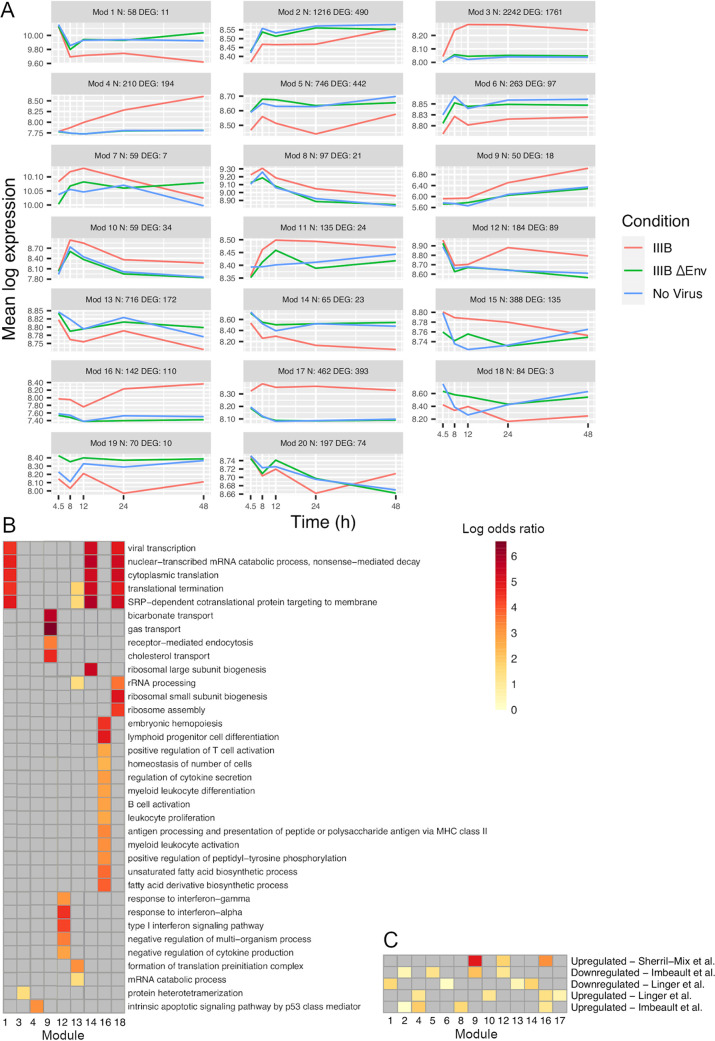
Weighted gene coexpression network analysis (WGCNA) of microarray data was used to group probes whose gene expression measurements correlated across samples into so-called modules (Mod). (A) WGCNA grouped 15,407 probes into 35 modules. Of these, 20 modules exhibited an average gene expression profile over time for IIIB-infected cells that significantly (FDR < 5%) differed from the relatively similar profiles for mock- and IIIBΔEnv-infected cells. (B) Gene ontology (GO) enrichment analysis was applied to the genes represented by the probes in each module to establish the degree to which each characteristic temporal gene expression pattern represents known functional categories. SRP, signal recognition particle; MHC, major histocompatibility complex. (C) Application of the WGCNA method to previously published primary T-cell data showed upregulation of similar groups of genes in both studies.

To identify and characterize infection-dependent temporal patterns of gene expression without fixed significance and fold change thresholds, we applied weighted gene coexpression network analysis (WGCNA) to group together probes whose gene expression measurements correlated across samples into so-called modules. WGCNA is an unsupervised method that does not take into account the time point or experimental condition represented by each sample. From our previously defined gene universe, WGCNA grouped 15,407 probes into 35 modules. Of these, 20 modules exhibited an average gene expression profile over time for IIIB-infected cells that significantly (FDR < 5% [see Materials and Methods]) differed from those for mock- and IIIBΔEnv-infected cells (Fig. 3A). A large proportion (4,108/7,443; odds ratio [OR] = 18.35; *P* < 10^−15^) of the probes comprising these 20 modules were among the 5,280 probes that detected significant (FDR < 5%) differential expression between IIIB- and mock- or IIIBΔEnv-infected cells in our time point-specific tests, indicating that these 20 modules represent most of the temporal gene expression patterns that are characteristic of the *ex vivo* CD4^+^ T-cell response to infection with HIV-1. Indeed, and as expected, substantial numbers of probes grouped into modules (e.g., modules 3, 4, 16, and 17) representing increased and sustained expression in cultures infected with IIIB.

We applied gene ontology (GO) enrichment analysis to the genes represented by the probes in each module to assess the degree to which each characteristic temporal gene expression pattern represents known functional categories (Fig. 3B). Immune cell activation and proliferation categories were enriched for genes upregulated from 4.5 h postinfection onwards (module 16). Innate immune response categories were enriched for genes upregulated later, from 24 h onwards (module 12). p53-mediated apoptosis was enriched for genes upregulated from 8 h, with upregulation increasing over time (module 4). Transport-related categories were enriched in a small group of upregulated genes with overall low absolute expression levels (module 9). Four modules with genes downregulated from 12 to 24 h postinfection substantially overlapped in terms of enriched GO categories that mostly related to translation but also related to viral transcription and nonsense-mediated decay (modules 1, 13, 14, and 18). For module 3, to which the highest number of genes grouped, only a single GO category, protein heterotetramerization, was enriched. This enrichment was due to differential expression of a number of histone genes.

**FIG 4:**
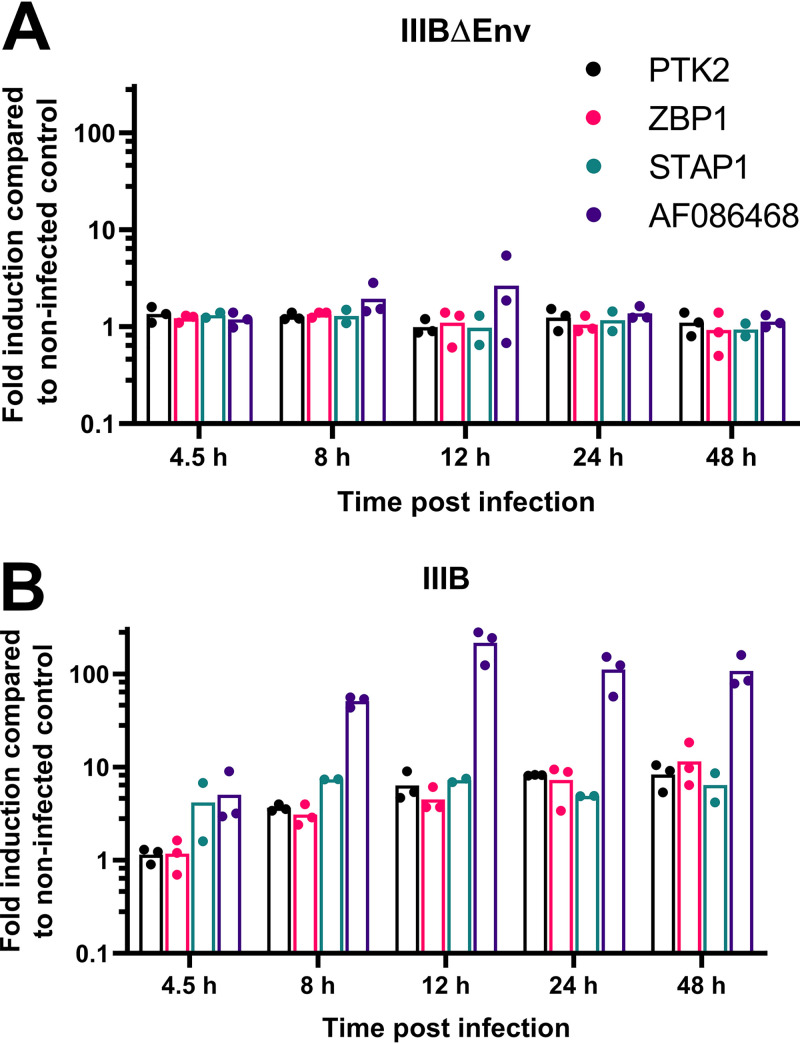
qPCR validation of gene expression changes. Primary CD4^+^ T cells were isolated, stimulated, and infected with either IIIBΔEnv (A) or IIIB (B) as described previously. At 4.5, 8, 12, 24, or 48 h postinfection, aliquots of cells were harvested, RNA was extracted, cDNA was generated, and the expression of PTK2, STAP1, ZBP1, and AF086468 was analyzed by qPCR. Results are expressed using the ΔΔ*C_t_* method (where *C_t_* is threshold cycle) and normalized to β-actin and GAPDH levels. Three donors were used and are represented as dots on the graph, with bars demonstrating the mean value. The difference in fold changes in gene expression between IIIB and IIIBΔEnv for each gene (excluding STAP1) at each time point was statistically significant (adjusted *P* value of <0.05, assessed using the unpaired Student *t* test, with individual variances computed for each comparison and *P* values adjusted using Holm-Šídák’s multiple-comparison test in Prism version 9.0.0) from 8 h onwards.

Application of the WGCNA method to previously published primary T-cell data showed upregulation of similar groups of genes (Fig. 3C). An upregulation of genes related to the type I IFN response has also been noted in multiple previous analyses of gene expression in CD4^+^ T cells isolated from viremic patients, though it should be recognized that the majority of CD4^+^ T cells are not, themselves, infected in this context (7, 18). The downregulation of genes related to transcription and translation has been identified in previous studies of HIV-1 infection (14, 15, 19). The previously identified Nef-mediated upregulation of genes involved in cholesterol metabolism was also recapitulated in our data (20). Ontology categories enriched among genes in module 9 have not prominently featured in previous studies of HIV-1 infection in either primary cells or cell lines. Quantitative real-time PCR (qPCR) was then used to verify the differential expression of 3 protein-coding genes (*PTK2*, *ZBP1*, and *STAP1*) that were identified as consistently upregulated in infected cells by microarray analysis and an expressed sequence tag (NCBI accession no. AF086468), the corresponding microarray probe for which had detected the highest upregulation of all probes in the data set (Fig. 4). These probes were not chosen based on the WCGNA; however, AF086468 and ZBP1 fall into module 9, PTK2 falls into module 12, and STAP1 falls into module 5.

### Host gene expression modulation is a shared feature of CXCR4- and CCR5-tropic viruses.

**FIG 5:**
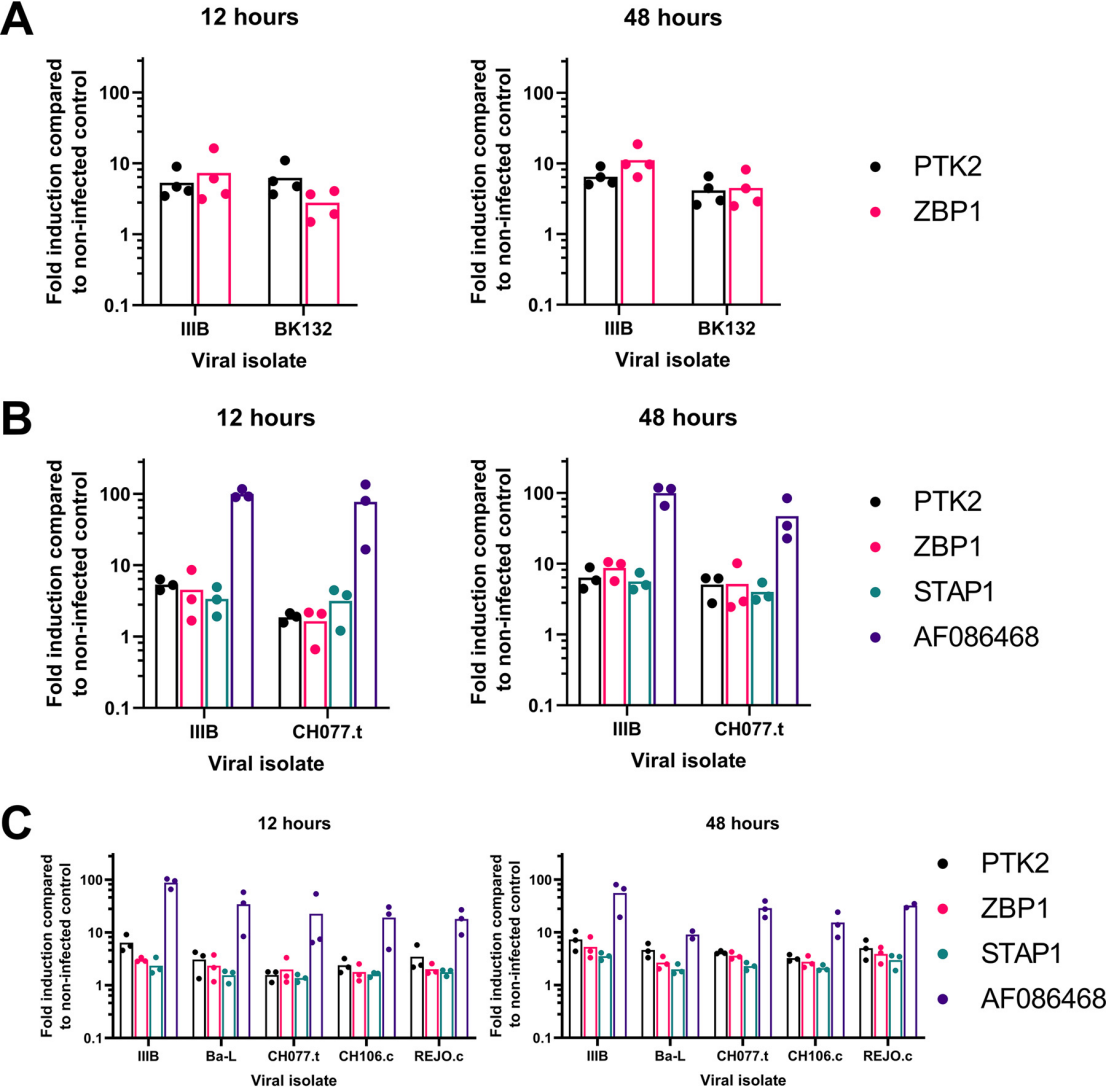
The induction of gene expression changes is conserved across a range of HIV-1 isolates. (A to C) Infections were repeated in total (A and B) or memory (C) CD4^+^ T cells, and changes to gene expression were assessed by qPCR at 12 or 48 h postinfection with the X4-tropic HIV-1 isolate BK132 (A), the dually tropic transmitted founder virus CH077.t (B and C), and the R5-tropic isolates Ba-L, CH106.c, and REJO.c (C). Dots represent values for individual donors, with bars showing the means of all values presented.

To ensure that the observed transcriptomic response was not idiosyncratic to IIIB, total CD4^+^ T cells were isolated from three donors and challenged with either IIIB, BK132 (an X4-tropic primary isolate of HIV-1), or CH077.t (a dual tropic transmitted founder virus). Gene expression in infected cells was analyzed by qPCR at 12 and 48 h postinfection and compared with that after mock infection; a response to all three viral strains was demonstrated (Fig. 5A and B).

Previous studies have shown that the principal target cells for HIV-1 infection *in vivo* are memory CD4^+^ T cells and that transmitted HIV-1 strains are predominantly CCR5 (R5) tropic (21, 22). For these reasons, CD4^+^ CD45RO^+^ memory T cells were isolated from three further donors and infected with either IIIB, Ba-L (a CCR5-tropic primary isolate of HIV-1), CH077.t, CH106.c, or REJO.c (the last two of which are CCR5-tropic transmitted founder viruses). RNA expression analyses confirmed a similar transcriptional response in memory CD4^+^ T cells to all viral strains tested (Fig. 5C).

### The rapid gene expression changes that occur by 12 h are interferon independent.

**FIG 6:**
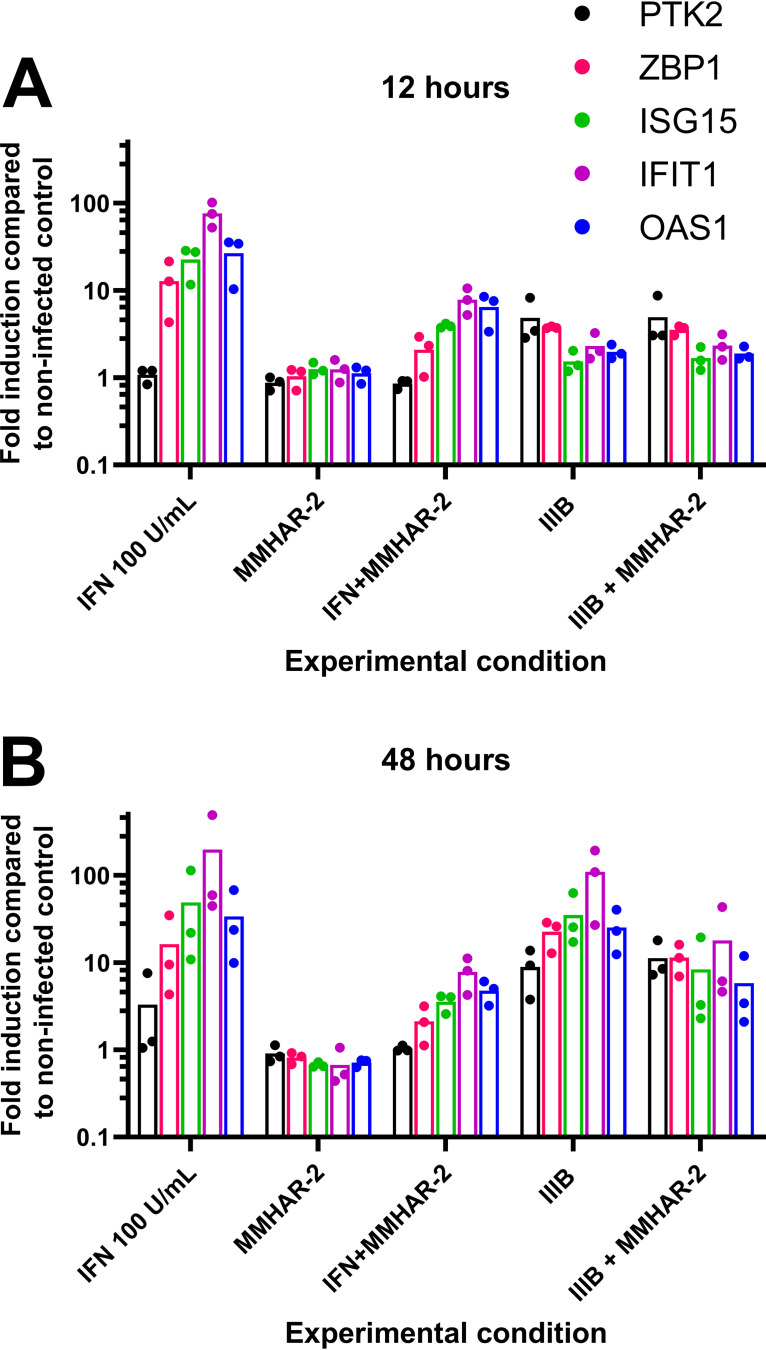
Type 1 IFN contributes to gene expression changes seen at 48, but not at 12 h, postinfection. Total CD4^+^ T cells were either treated or not treated with type 1 IFN receptor-blocking antibody MMHAR-2 1 h prior to either treatment with IFN-α or spin infection with IIIB. Levels of expression of PTK2, ZBP1, IFN-stimulated gene 15 (ISG15), IFN-induced protein with tetratricopeptide repeats 1 (IFIT1), or 2′-5′-oligoadenylate synthetase 1 (OAS1) were measured at 12 (A) or 48 (B) h postinfection and compared to their expression in mock samples. Results are expressed using the ΔΔ*C_t_* method normalized to β-actin and GAPDH levels. A minimum of three donors were used and are represented as dots on the graph, with bars demonstrating the mean values.

The analysis described above identified an upregulation of ISGs from 24 to 48 h postinfection (Fig. 3B). This observation agreed with previous studies of the transcriptomic response of primary CD4^+^ T cells both *in vivo* and *ex vivo* (9, 12, 13, 18). It was not, however, possible to identify increased expression of type I IFNs themselves by microarray analysis, qPCR, or bioassay (data not shown for the last two). Accordingly, to examine the possible contributions of IFN and IFN-induced signaling to ISG expression changes at 12 and 48 h postinfection, IIIB infections of CD4^+^ T cells were carried out in the presence or absence of an antibody specific for the type I IFN receptor (MMHAR-2) that efficiently blocks IFN-induced signaling, and RNA levels of a subset of ISGs (ISG15, IFN-induced protein with tetratricopeptide repeats [IFIT1], and 2′-5′-oligoadenylate synthetase 1 [OAS1]) were then monitored by qPCR. The addition of MMHAR-2 had a negligible impact on HIV-1-induced gene expression at 12 h but partially suppressed induction when measured at 48 h (Fig. 6). We interpret these data as revealing that the HIV-1-induced expression changes seen at 12 h occur independently of IFN but that IFN contributes to the activation of ISG expression at later time points.

### The HIV-1 accessory protein Vpr initiates cellular RNA expression changes during the initial hours of infection.

**FIG 7:**
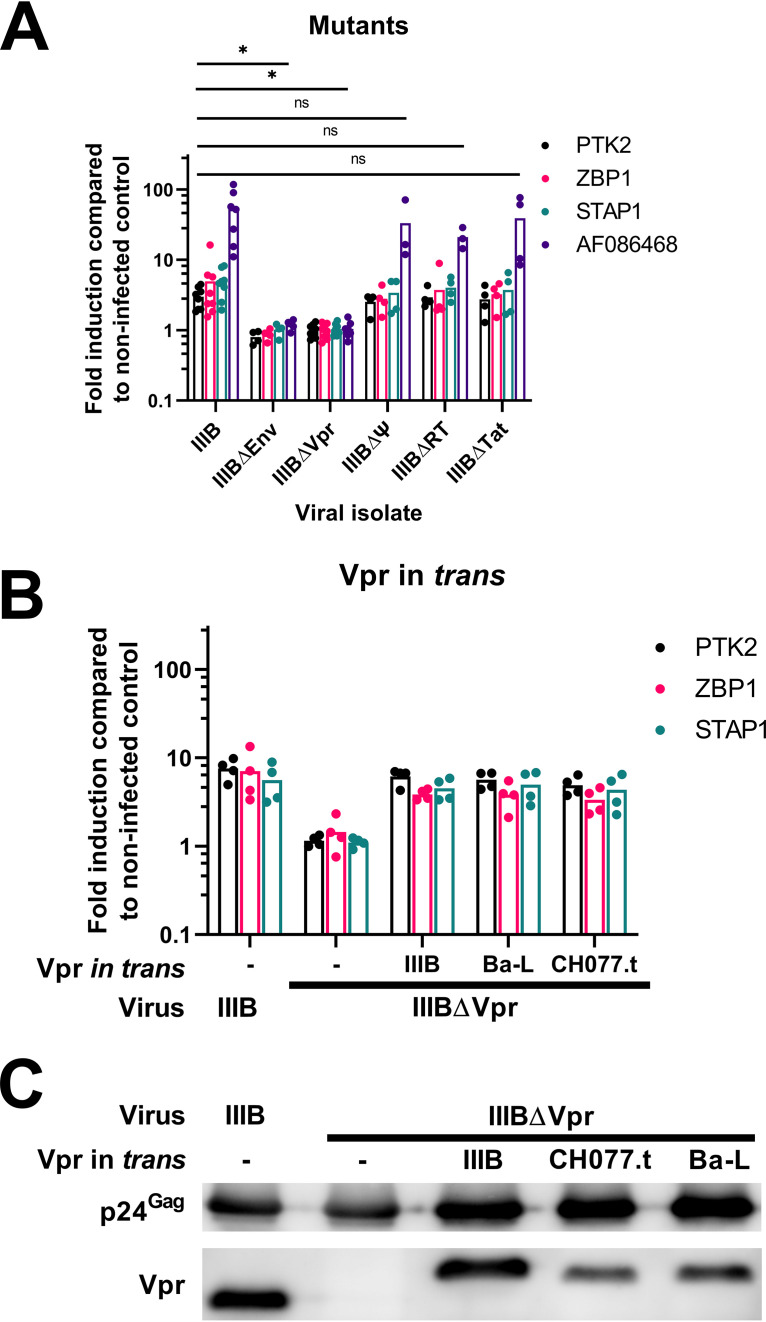
HIV-1 Vpr triggers the gene expression changes early postinfection. (A) Total CD4^+^ T cells were isolated and stimulated as described previously before spin infection with either IIIB, IIIBΔEnv, IIIBΔΨ, IIIBΔRT, IIIBΔTat, or IIIBΔVpr, and gene expression was assessed by qPCR 12 h postinfection with at least 3 donors per condition. Gene expression changes for modified viruses were compared to those for IIIB using unpaired Student *t* tests, with individual variances computed for each comparison and *P* values adjusted using Holm-Šídák’s multiple-comparison test in Prism version 9.0.0. (B) Restoring Vpr to IIIBΔVpr viral particles by cotransfecting a plasmid containing Vpr from the IIIB, Ba-L, or CH077.t isolate in *trans* during viral production restored gene expression changes at 12 h postinfection. (C) The presence of Vpr in these viral particles was confirmed by immunoblotting.

The observed early transcriptional response was likely to have been stimulated by the constituents of incoming virions. In particular, genomic RNA, the DNA products of reverse transcription, and the capsid lattice are known to be sensed by pattern recognition receptors and to stimulate innate immune signaling (23–28). For this reason, a series of mutated viruses were used to challenge primary CD4^+^ T cells, and differential RNA expression was monitored at 12 h by qPCR. Specifically, IIIBΔEnv (Fig. 1B) served as the negative control, IIIBΔΨ has a disrupted guide RNA (gRNA) packaging signal and encapsidates reduced levels of gRNA (29, 30), IIIBΔRT carries an inactivated reverse transcriptase (RT) gene and is unable to synthesize viral cDNA, IIIBΔTat lacks the transcriptional transactivator *tat* and is severely defective for viral transcription (31), and IIIBΔVpr lacks the accessory protein Vpr, an ∼14-kDa nonstructural protein that is selectively incorporated into nascent virions via an interaction with the p6 region of p55^Gag^ (32–34) but whose functions in the context of virus replication are not fully understood (35–37). Disruption of gRNA packaging, RT, or Tat had negligible effects on virus-induced alterations to gene expression, but the removal of Vpr abrogated the cellular response (Fig. 7A).

It was formally possible that the loss of the inductive capability of IIIBΔVpr was due to the mutation of viral sequence rather than the loss of the Vpr protein itself. To address this, IIIBΔVpr was complemented in the cultures used to produce viral stocks by cotransfection with Vpr expression vectors corresponding to three different HIV-1 isolates. In each case, the activation of host gene expression at 12 h in CD4^+^ T cells was efficiently restored to levels comparable to those of wild-type virus (Fig. 7B), demonstrating that the initiating signal for the transcriptomic changes seen in primary CD4^+^ T cells is Vpr dependent. Lastly, the efficient incorporation of these Vpr proteins in *trans* into IIIBΔVpr particles was confirmed using purified virions and immunoblotting (Fig. 7C).

### Transcriptomic analysis of Vpr-regulated genes.

**FIG 8:**
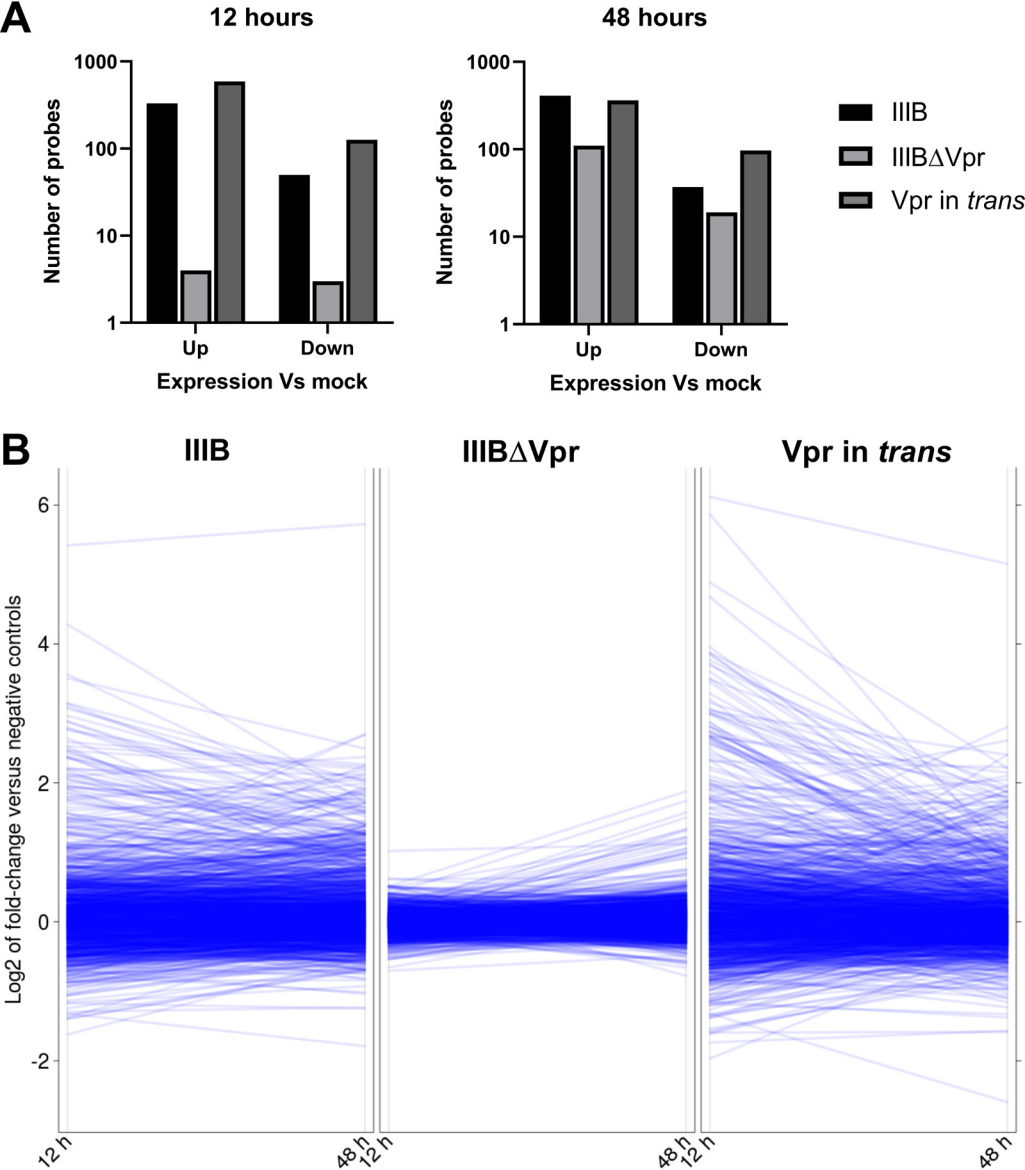
Vpr has a global effect on the transcriptome of CD4^+^ T cells early postinfection. (A) Total CD4^+^ T cells were spin infected with either IIIB, IIIBΔVpr, or IIIBΔVpr plus Vpr. Cells were harvested at 12 or 48 h postinfection, and gene expression was assessed using Illumina’s BeadArray HT12v4. The numbers of probes detecting >2-fold changes in expression (FDR < 5%) from mock infection are demonstrated. (B) Three contrasting composites of line plots illustrating the absence of large gene expression differences at 12 h and, to a lesser degree, at 48 h postinfection with IIIBΔVpr relative to mock-infected cells (center panel titled “IIIBΔVpr”), in contrast to infection with wild-type IIIB (left) or Vpr in *trans* (right). The set of 1,303 probes for which the relative expression values are plotted is the same as in Fig. 1B.

**FIG 9:**
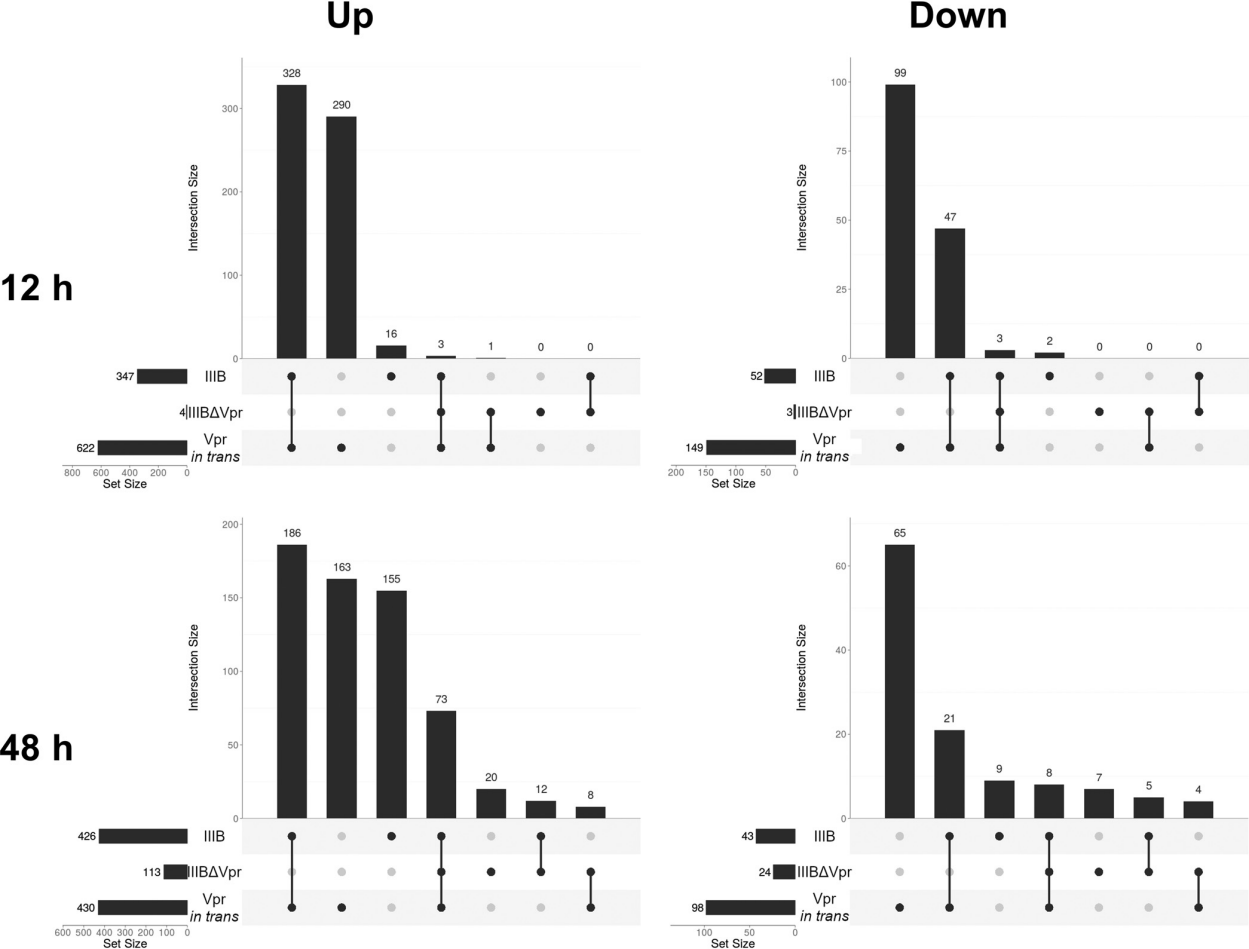
Restoring Vpr to IIIBΔVpr virus particles recapitulates the gene expression changes seen in wild-type virus infection. As in Fig. 2, horizontal bars visualize the numbers of probes detecting differential gene expression of 2-fold or more (FDR < 5%) 12 h (top) and 48 h (bottom) postinfection with either IIIB, IIIBΔVpr, or IIIBΔVpr with Vpr in *trans* relative to expression in mock-infected cells. Numbers of probes detecting upregulation (left) versus downregulation (right) are shown separately. Vertical bars illustrate the sizes of all possible mutually exclusive subsets that can be formed via the intersection of these three sets of probes.

To more broadly determine the role of Vpr, repeat experiments with primary CD4^+^ T cells were analyzed using Illumina’s BeadArray HT12v4 (Fig. 8). At the 12-h time point, almost all differential gene expression seen with the wild-type virus was lost in the absence of Vpr but restored when Vpr was provided in *trans*. At 48 h postinfection, a low level of differential expression was observed with IIIBΔVpr; however, the response remained far more limited than in the presence of Vpr (Fig. 8A and B). As expected, at 12 h postinfection, there was a large degree of overlap in the identities of cellular genes upregulated following challenge with IIIB and IIIBΔVpr plus Vpr (in *trans*), additionally confirming that virion-packaged Vpr was responsible for inducing the observed transcriptome response (Fig. 9). At 48 h postinfection, a larger number of genes was seen upregulated following IIIB infection but not after infection with IIIBΔVpr plus Vpr (in *trans*). Previous studies have suggested that Vpr may play a role in either eliciting or suppressing a type I IFN response following HIV-1 infection (38–41).

## DISCUSSION

Studying the transcriptomic response of cells to viral infection in terms of the genes that may promote or impede infection can provide new insights into host-pathogen interactions. While there are a number of prior studies for HIV-1, few have been conducted with primary CD4^+^ T cells, and none of these have interrogated the first 24 h of infection. We therefore sought to expand on published data by examining transcriptional responses in primary CD4^+^ T cells across a range of early time points using X4- and R5-tropic viral strains. By doing so, we showed for the first time that changes to cellular gene expression in the early hours following infection are triggered by Vpr.

Our study identified patterns of differential gene expression that were broadly similar to those found in previously published data sets (11, 13–15, 19, 42, 43). In particular, we documented an upregulation of genes associated with the innate immune response, cytokine production, and apoptosis (13, 42, 43). The downregulation of genes related to transcription and translation that we observed has previously been reported both in primary CD4^+^ T cells and T-cell lines (14, 15, 19, 42). In addition, of particular interest, an overrepresentation of histone genes led to protein heterotetramerization being identified in our gene ontology enrichment analysis. The confirmation of transcriptomic responses to infection in memory as well as total primary CD4^+^ T cells and with multiple X4- and R5-tropic viral strains further validated the generality of our findings.

One previous study has provided a detailed examination of the early (first 24 h) transcriptional response of SupT1 cells to HIV-1 infection (15). The study identified groups of genes with different temporal patterns of expression, many of which we similarly observed, for example, genes that were downregulated throughout infection, genes with rising levels of upregulation across successive time points, and genes that were specifically upregulated later in infection. Though direct comparison of the identities of genes in these similar groups was not possible owing to data inaccessibility, a minimum of overlap might be anticipated due to differences in cell types and the methods used for analysis (13). With regard to overall trends, both our study and this previous work noted initial downregulation of genes associated with transcription and translation and later upregulation of genes involved in immune signaling.

Our most striking finding was the dependence of initial gene expression changes on Vpr. Arguably the least well understood of the HIV-1 accessory proteins, Vpr has previously been associated with a number of activities, most prominently the arrest of the cell cycle in G_2_/M (44–47). It has also been linked to the modulation of innate immune signaling and enhanced gene expression from HIV-1 and cellular promoters, both activities that potentially impact cellular transcriptional profiles. With respect to signaling, multiple studies have examined the role of Vpr in NF-κB signaling pathways. However, they disagree as to whether Vpr promotes (48, 49) or inhibits (50–52) NF-κB signaling and at which point in the pathway this occurs (48, 50, 52). Equally, in the context of primary CD4^+^ T-cell infection with replication-competent virus, other viral proteins have been shown to play a role (14). In addition, a previous study of the transcriptional response of monocyte-derived macrophages (MDMs) to Vpr suggested an upregulation of genes associated with innate immunity (53). However, this is in apparent contrast with other studies showing that HIV-1 evades stimulation of the type 1 IFN response in MDMs and that Vpr restricts the translocation of IRF3 to the nucleus in monocytic cell lines (52, 54).

Multiple previous studies have also shown a direct impact of Vpr on transcription from viral and cellular promoters. While a variety of transcription factor binding sites have been implicated in this process, most studies used transient transfection of cell lines to demonstrate these effects. More compelling are recent studies showing Vpr-mediated increases in viral transcript levels from integrated or unintegrated viral DNA (55, 56). Although the heterogeneity of prior studies makes direct comparisons with our results challenging, our findings, overall, demonstrate a central role for Vpr in the broad modulation of cellular mRNA expression early postinfection in a pattern that has not previously been described.

Vpr has been shown to co-opt the cellular E3 ubiquitin ligase consisting of cullin 4A (CUL4A), DNA damage binding protein 1 (DDB1), and CUL4A- and DDB1-associated factor 1 (DCAF1) to degrade cellular targets (57–62). Recent evidence supports the hypothesis that many of these are recruited by interaction with their DNA binding domains (63–65). One recent extensive proteomic study used multiple mass spectrometry approaches to evaluate infection with viruses in the presence or absence of Vpr and identified many new primary protein targets of Vpr, a proportion of which have the potential to directly modify transcription (65). Other publications have shown DCAF1-dependent degradation of proteins capable of epigenetic modification, including class I histone deacetylases (66, 67), ZGPAT (Zinc finger CCCH-type and G-patch domain containing) (68), and TET2 (10-11 translocation) (69). Two groups have also recently shown Vpx (but not HIV-1 Vpr)-mediated degradation of members of the human silencing hub (HUSH) complex, which resulted in reactivation of HIV-1 in a latency model and more rapid replication kinetics in spreading infection (70, 71). These studies may therefore provide clues as to how Vpr may promote the transcriptional reprogramming of genome-wide gene expression and will help direct future work examining molecular mechanisms. It is also not yet clear whether the Vpr-mediated modification of the cellular transcriptome is beneficial or detrimental to HIV-1 replication, and answering this question represents a further priority.

A notable (though experimentally necessary) limitation of our study was the relatively high viral inoculum used to generate a synchronously infected population of cells where transcriptomic responses could be monitored during the initial phases of infection. Whether cells are naturally exposed to such high levels of viral particles *in vivo* is not clear, though high multiplicities of HIV-1 infection have been reported in the context of lymphoid tissue (72). Equally, it was not possible to show how many cells identified as p24^Gag^ positive at 3 h postinfection were productively infected. However, experiments using ΔVpr virus complemented with Vpr during viral production (Fig. 8), together with a panel of replication-deficient viruses (Fig. 7), demonstrated that early changes to transcription were a consequence of virion-associated Vpr, regardless of whether infection was ultimately productive. Future work using methodologies at the single-cell resolution will seek to provide additional insights into these observations, though the general correspondence between our findings and the patterns of transcription described in previous studies provides important overall confirmation.

The *in vivo* importance of Vpr is clearly demonstrated by its conservation among lentiviral lineages, attenuated infection in its absence, and reversion of disrupted alleles over the course of infection in both humans and animals (73–79). However, the underlying basis for this importance remains elusive. Our demonstration that encapsidated Vpr has a profound effect on the CD4^+^ T-cell transcriptome in the earliest phases of HIV-1 infection therefore casts new light on previous transcriptomic analyses of HIV-1 infection and highlights a new and important aspect of the interaction between HIV-1 and its host. Further studies to determine whether this property is conserved among the Vpr proteins from other lentiviral lineages would be of interest.

## MATERIALS AND METHODS

### HIV-1 molecular clones and plasmids.

The wild-type HIV-1 proviral clone pIIIB and its *env*-deficient derivative pIIIBΔEnv have been described previously (80–82). The pIIIBΔΨ mutant was generated by deleting a portion of SL2 and the entire SL3 region (i.e., between nucleotides 750 and 788, in accordance with the work of Aldovini and Young [29]). Point mutations were introduced into the IIIB reverse transcriptase catalytic site (YMDD338-341YAAA) to generate the pIIIBΔRT mutant. pIIIBΔVpr and pIIIBΔTat were generated by introducing 2 stop codons at the beginning of the *vpr* or *tat* open reading frames with point mutations (G5629T and G5632T for *vpr* and ATCATC5855-5860TAGTAG for *tat*). The transmitted founder HIV-1 molecular clones CH077.t, CH106.c, and REJO.c were kindly provided by Beatrice Hahn (83). The HIV-1 Ba-L and BK132 virus isolates were obtained from the NIH AIDS Reagent and Reference Program (generously provided by Suzanne Gartner, Mikulas Popovic, and Robert Gallo [84] and Nelson Michael [85], respectively).

Amino-terminally Flag-tagged *vpr* genes from NL4-3, CH077.t, and Ba-L were amplified by PCR from proviruses (NL4-3, CH077.t) or by reverse transcription-PCR from Ba-L-infected CCR5 Jurkat cells and cloned into pcDNA3.1+. pcDNA3.1+Tat was made by PCR amplification of IIIB Tat from pcTat (86) and cloning into pcDNA3.1 using HindIII and XbaI and was a kind gift from Chad Swanson.

### Cells and cell culture.

Human primary CD4^+^ T cells were obtained from peripheral blood mononuclear cells (PBMCs) of healthy volunteer donors (approved by the Guy’s research ethics committee [reference no. 03/02/06]). Briefly, PBMCs were isolated using Lymphoprep (Axis-Shield), and total or memory CD4^+^ T cells were then isolated from the remaining cells using CD4^+^ T-cell isolation kit II or the memory CD4^+^ T-cell isolation kit, respectively (Miltenyi Biotec). The purity of isolated cell populations was always >90%, as judged by flow cytometric analysis of specific markers (CD4 and CD3 or CD4 and CD45RO for total or memory T cells, respectively) (see Fig. S1 in the supplemental material). CD4^+^ T cells were cultured in Roswell Park Memorial Institute (RPMI) 1640 medium (Gibco) supplemented with 10% autologous human serum and stimulated for 40 h with anti-CD3 and anti-CD28 antibodies (BD Biosciences; final concentration, 1 to 2 μg/ml) prior to infection, and the antibodies were readded upon medium changing after infection. Human 293T cells were maintained in complete Dulbecco’s modified Eagle’s medium (DMEM) (Gibco) plus 10% fetal bovine serum (FBS), and Jurkat T cells were grown in RPMI 1640 medium supplemented with 10% FBS.

10.1128/mBio.01369-21.1 FIG S1 Representative plots of gating strategy for assessment of purity of isolated CD4^+^ T cells. Following isolation from PBMCs, aliquots of CD4^+^ T cells were stained using either allophycocyanin (APC)-conjugated anti-CD4 and *R*-phycoerythrin (PE)-conjugated anti-CD3 (lower row) or corresponding isotype control antibodies, and CD4 and CD3 expression was measured by flow cytometry (right). Download FIG S1, TIF file, 0.4 MB. Copyright © 2021 Bauby et al. 2021 Bauby et al. https://creativecommons.org/licenses/by/4.0/ This content is distributed under the terms of the Creative Commons Attribution 4.0 International license.

### Viral production.

Wild-type or mutant HIV-1_IIIB_, HIV-1_CH077.t_, HIV-1_CH106.c_, or HIV-1_REJO.c_ were produced by standard polyethylenimine (PEI) transfection of 293T-cell monolayers. IIIBΔTat was produced by cotransfection of the corresponding provirus and pcDNA3.1+Tat at a ratio of 1:0.2. To test the effect of Vpr proteins from other strains, pIIIBΔVpr provirus was cotransfected with pcDNA3.1+Vpr expression plasmids at a ratio of 1:0.25. The culture medium was changed at ∼6 h, and the virus-containing supernatant was harvested at ∼36 h. HIV-1_Ba-L_ and HIV-1_BK132_ were grown in CCR5-expressing Jurkat cells as previously described (87).

Viruses were filtered and incubated with 20 U/ml RQ1 DNase (Promega) and 10 mM MgCl_2_ for 3 h at 37°C. Viruses were then purified by ultracentrifugation through a sucrose cushion (20%, wt/vol; 75 min of 4°C at 145,370 × *g* using a Sorvall Surespin630 rotor), resuspended in RPMI 1640 medium without serum, and stored in aliquots at −80°C. Viral particles were normalized according to the results of an HIV-1 p24^Gag^ enzyme-linked immunosorbent assay (ELISA) (Perkin Elmer).

### Immunoblot analysis.

Virus particles containing 250 ng of p24^Gag^ were lysed in sample buffer (200 mM Tris-HCl, pH 6.8, 5.2% SDS, 20% glycerol, 0.1% bromphenol blue, 5% β-mercaptoethanol), resolved by SDS-PAGE, and analyzed by immunoblotting using primary antibodies specific for p24^Gag^ (no. 24-2) (88) or Vpr (with Vpr-specific polyclonal antiserum raised in rabbits following immunization with a purified fusion protein comprising the 71 amino-terminal residues of HIV-1 Vpr appended to the carboxy terminus of maltose binding protein [UP1192] [32]), followed by secondary horseradish peroxidase-conjugated anti-mouse or anti-rabbit immunoglobulin antibodies, respectively, and chemiluminescence (ECL+ Western blotting substrate; Pierce).

### HIV-1 infection.

Infections were initiated by spin infection of the cells with HIV-1 particles (0.5 to 1 μg/ml p24^Gag^) for 2 h at 2,000 × *g* and at 30°C (89). The cells were then incubated at 37°C for 1 h prior to extensive washes in PBS. Fresh and prewarmed medium (containing anti-CD3 and -CD28 antibodies) was added, and the cells were placed back at 37°C for the indicated incubation times, prior to RNA extraction.

The percentage of cells in which the virus had efficiently entered was routinely analyzed by p24^Gag^ intracellular staining of cells at 3 h postinfection (i.e., at the time of the PBS washes) and flow cytometry (FACSCalibur; BD Biosciences). Briefly, the cells were washed in PBS, incubated for 10 min in prewarmed trypsin to remove surface-associated virion particles, fixed, permeabilized (IntraStain kit; Dako), and stained with a p24^Gag^-specific antibody (KC57-RD1; Beckman Coulter) (90). The percentage of p24^Gag^-positive cells was typically >70% for IIIB (Fig. S2). This antibody does not recognize p24^Gag^ from HIV-1_CH106.c_ or HIV-1_REJO.c_ and was therefore not used on memory T-cell infections with these viruses. For experiments assessing the role of IFN in gene expression, a monoclonal antibody specific for the type 1 IFN receptor (MMHAR-2; Invitrogen) was added to the indicated wells at a final concentration of 10 μg/ml 1 h before infection. For conditions treated with IFN-α (Intron A; Merck, Sharpe & Dohme Corp.), this was added to a final concentration of 100 U/ml at the start of the infection.

### Microarray data generation and analysis.

Primary CD4^+^ T cells (2 × 10^5^ to 2 × 10^6^) were harvested at the indicated times postinfection. RNA was isolated using the miRNeasy kit with on-column DNase treatment (Qiagen). One hundred to 500 ng RNA was used for cRNA probe preparation using the Illumina TotalPrep RNA amplification kit (Ambion) according to the manufacturer’s instructions. The probes were hybridized on Illumina HT12v4 bead arrays by following the manufacturer’s standard hybridization and scanning protocols.

Raw signals, detection *P* values, bead numbers, and bead-level standard errors were exported for regular and control probes from GenomeStudio (Illumina). Data were imported into R using the Bioconductor bead array and illuminaHumanv4.db packages. For the purposes of preprocessing, normalization, and removal of probes that did not detect a signal above background, we used a larger data set than is presented here. All arrays used for this analysis have been deposited in GEO as described below. The raw signal intensity distributions and the signal intensities for control probes (labeling, hybridization, housekeeping gene, and negative controls) were nominal for all but one array; signal intensities for the negative control and regular probes alike were elevated for array 9031292066_A. Signal intensities were background corrected, quantile normalized, and log_2_ transformed using the limma (91) implementation of the neqc method Shi (92). Unsupervised hierarchical clustering of the normalized data did not identify any outliers but rather a systematic array batch effect (November 2012 versus March 2013 batches) so that batch always was included as a covariate in subsequent linear models of data comprising arrays from both batches. Array 9031292066_A, in particular, belonged to an unremarkable cluster of other arrays from the same batch assaying the same cell type and, therefore, was retained for analysis. Multidimensional scaling analysis of the array measurements for *XIST* and for highly expressed genes on chromosome Y exhibiting a gender-corresponding, bimodal, expression-level distribution (*KDM5D*, *TXLNG2P*, *EIF1AY*, *RPS4Y1*, *RPS4Y2*, *UTY*, *USP9Y*, *PRKY*, *ZFY*) bipartitioned arrays into two distinct gender-specific clusters. Cluster membership always corresponded to the annotated proband (donor) gender of the sample; i.e., no sample swaps became apparent.

Probes that did not detect a signal significantly above the global (for all arrays and all probes) signal median (considered the background) under any of the distinguished experimental conditions were excluded from analysis. Specifically, we fitted three zero intercept linear models that included batch as a covariate and the following experimental factors: (i) cell type (total versus memory CD4^+^ T cells), (ii) cell type and infection state (infected with HIV-1 versus mock infected with IIIBΔEnv or no virus), and (iii) cell type, infection state, and time point after infection. Separately for each model and experimental condition, probes were tested for above-background signals (limma treat function [93] with LFC, which is the global median). Probes detecting an above-background signal in any of the conditions of any of the models at a significance threshold FDR of <20% were retained for analysis.

Array-quality weights were inferred with the limma arrayWeightsSimple method (94) using a model with batch and proband as covariates and cell type, infection status, and time point as the experimental factors. The array weights were used in all subsequent applications of the limma lmfit model fitting function. All linear models addressing biological questions (Table 1) also included proband as a covariate to account for interindividual differences in gene expression and/or in probe hybridization efficiency due to genetic variants from the probe sequence. For each model, multiple-testing correction was carried out globally using the Benjamini-Hochberg method for controlling the FDR (limma decideTests with method set as “global” and adjust.method set as “BH”). The bar chart visualizations of the sizes of sets of differentially expressed genes and their intersections were generated with a customized version of UpSetR v1.0 (95).

**TABLE 1:** Linear models used in bioinformatic analysis of microarray results

Model no.	Included samples	Modeled factors and levels	Coefficient comparison(s)
1	All either IIIBΔEnv- or mock-infected samples	Proband and virus (IIIBΔEnv or no virus) by time point (4.5, 8, 12, 24, or 48 h) and cell type (total or memory CD4^+^ T cells) and batch (November 2012 or March 2013)	IIIBΔEnv vs no virus, separately for each time point
2	Total CD4^+^ T cells from the same three donors under all conditions (virus and time point) belonging to the same batch (November 2012)	Proband and virus (IIIB, IIIBΔEnv, or no virus) by time point (4.5, 8, 12, 24, or 48 h)	2a: separately for each virus, all pairwise comparisons of a time point vs an earlier time point
			2b: separately for each time point, IIIB and IIIBΔEnv vs no virus
3	All total CD4^+^ T-cell samples infected with either IIIB, IIIB ΔEnv, or no virus	Proband and virus (IIIB or negative control) by time point (4.5, 8, 12, 24, or 48 h) and batch	Separately for each time point, IIIB vs the negative control
4	All total CD4^+^ T-cell samples 12 or 48 h postinfection with either IIIB, IIIBΔVpr, IIIBΔVpr plus Vpr, IIIBΔEnv, or no virus	Proband and virus (IIIB, IIIBΔVpr, IIIBΔVpr plus Vpr, or negative control) by time point (12 or 48 h) and batch	Separately for each time point, IIIB, IIIBΔVpr, and IIIBΔVpr plus Vpr vs the negative control

### WGCNA.

The weighted gene coexpression network analysis (WGCNA) tool (96) was utilized. Since WGCNA employs an unsupervised algorithm, unwanted sources of variation were removed prior to its application. Specifically, the effects of individual proband (donor) were removed using the ComBat function of the sva package (97). The data set comprised more control than infected samples. To avoid a disproportionate contribution of control samples to gene module definition, the data were separately analyzed in two parts: (i) infected samples and uninfected controls and (ii) infected samples together with ΔEnv-challenged samples separately. The full WGCNA workflow was then applied for identification of gene expression modules. First, adjacency matrices were generated from probe-level expression data using the pickSoftThreshold and adjacency functions. These matrices were converted to distance matrices before construction of topological overlap matrices (TOM) with the TOMsimilarity function. The resultant TOMs (one generated using the uninfected controls and one using the ΔEnv controls) were scaled in order to equate the 95th percentiles, and a single consensus TOM was generated by taking the minimum topological overlap of the two input TOMs for all pairwise probe comparisons. This allowed an “average” gene expression network of the two control conditions (uninfected controls and ΔEnv controls) to be captured. The resultant TOM was used for hierarchical clustering prior to module definition via the cutTreeDynamic function/algorithm (96). Per-sample-per-module summarizations were generated via principal-component analysis (PCA), using the first eigenvector (also known as the eigengene). Modules with a pairwise Pearson correlation coefficient of >0.75 were combined.

To identify modules of interest, eigengenes were tested for significant association (FDR < 0.05) with infection using a linear model. This allowed identification of gene modules which specifically showed regulation in response to HIV-1 infection. Identified modules were tested for enrichment of gene ontology biological processes using the R interface of the g:Profiler toolkit (98) with “strong” hierarchical filtering and a significance threshold of set counts and sizes (SCS) of <0.05. Module enrichment for genes differentially expressed in previously published analyses of HIV infection was assessed via the hypergeometric test with a significance threshold FDR of <0.05. Previously published transcriptomic analyses of primary CD4^+^ T-cell infection with HIV-1 were utilized (12–14). Imbeault et al.(12) analyzed gene expression changes via exon array in HIV-1-infected cells relative to uninfected bystander cells at 24, 48, and 72 h postinfection. Genes differentially regulated during at least one time point, as reported by the authors, were used for enrichment analysis. Sherrill-Mix et al. (13) performed RNA sequencing (RNA-seq) of infected and uninfected cells 48 h postinfection; these data were reanalyzed as detailed below [see “Reanalysis of RNA-seq data by Sherrill-Mix et al. (13)”]. Langer et al. (14) compared levels of gene expression in primary CD4^+^ T cells either mock infected or infected with one of three different wild-type HIV-1 isolates using total RNA-seq. Differential gene expression data as detailed by the authors were obtained via personal communication, and differentially expressed genes were identified using a significance threshold FDR of <0.05.

### Reanalysis of RNA-seq data by Sherrill-Mix et al. (13).

The read data in FASTQ format were obtained from the Sequence Read Archive under accession no. SRP055981. Reads were adapter trimmed and quality and length filtered using TrimGalore v0.4.1 (http://www.bioinformatics.babraham.ac.uk/projects/trim_galore/) and Cutadapt v1.9.1 (99) with default parameters. Reads were aligned to the human genome (GRCh38 plus Ensembl v84 plus dbSNP v144) using the splice- and SNP-aware aligner HiSat2 v2.0.3-beta (100). Gene-level read count data were generated with GenomicAlignments and GenomicFeatures (101) with Gencode annotation v24 (Ensembl v83). Significantly (FDR < 10%) differentially expressed genes (DEGs) between the HIV_89.6_-1-infected (*n* = 3) and the mock-infected (*n* = 2) CD4^+^ T-cell samples (single donor) were identified using DESeq2 v1.10.1 (102) and, separately, using edgeR v3.12.1 (for log transformation with voom) (103) and limma v3.26.9 (104). DEGs concordantly called and quantified to have an absolute log_2_ fold change of >1 by both analysis methods were used as gene sets (upregulated, *n* = 913; downregulated, *n* = 324) in subsequent enrichment analyses.

### qPCR analysis.

cDNA was generated from 250 to 500 ng RNA by random priming using the high-capacity cDNA archive kit (Applied Biosystems) and analyzed by quantitative real-time PCR (qPCR) using TaqMan gene expression assays (Applied Biosystems) specific for human *β-actin* (Hs99999903_m1), glyceraldehyde-3-phosphate dehydrogenase (*GAPDH*; Hs99999905_m1), protein tyrosine kinase 2 (*PTK2*; Hs00178587_m1), Z-DNA binding protein 1 (*ZBP1*; Hs00229199_m1), signal-transducing adaptor family member 1 (*STAP1*; Hs00201585_m1), IFN-stimulated gene 15 (*ISG15*; Hs00192713_m1), IFN-induced protein with tetratricopeptide repeats 1 (*IFIT1*; Hs00356631_g1), and 2′-5′-oligoadenylate synthetase 1 (*OAS1*; Hs00973637_m1). Expression of AF086468 was analyzed using the following primers and probe: forward primer 5′-CTGATGGGGCCACGCTC, reverse primer 5′-GCAAAGCGACCCCAGGAC, and probe 5′-FAM-TTCTGAGGATTGCCAGGGAAACAGGAGGTGC-TAMRA, where FAM is 6-carboxyfluorescein and TAMRA is 6-carboxytetramethylrhodamine. The concentration for both primers was 900 nM, and that for the probe was 250 nM. Triplicate reactions were run according to the manufacturer’s instructions using an ABI Prism model 7900HT sequence detection platform, and the RQ software (Applied Biosystems) was used for subsequent analysis. For relative quantification, samples were normalized for both *GAPDH* and *β-actin* mRNA expression.

### Statistics.

Differences in levels of expression of specific genes between conditions measured by qPCR were assessed for statistical significance using an unpaired Student *t* test, with individual variances computed for each comparison, and *P* values were adjusted using Holm-Šídák’s multiple-comparison test. Calculations were performed in Prism version 9.0.0.

### Data availability.

The microarray data are available under GEO accession no. GSE166375.

## References

[1] Anderson HA, Chen Y, Norkin LC. 1996. Bound simian virus 40 translocates to caveolin-enriched membrane domains, and its entry is inhibited by drugs that selectively disrupt caveolae. Mol Biol Cell 7:1825–1834. doi:10.1091/mbc.7.11.1825. 8930903 PMC276029

[2] Mercer J, Helenius A. 2008. Vaccinia virus uses macropinocytosis and apoptotic mimicry to enter host cells. Science 320:531–535. doi:10.1126/science.1155164. 18436786

[3] Norkin LC. 2001. Caveolae in the uptake and targeting of infectious agents and secreted toxins. Adv Drug Deliv Rev 49:301–315. doi:10.1016/s0169-409x(01)00143-0. 11551401

[4] Pelkmans L, Fava E, Grabner H, Hannus M, Habermann B, Krausz E, Zerial M. 2005. Genome-wide analysis of human kinases in clathrin- and caveolae/raft-mediated endocytosis. Nature 436:78–86. doi:10.1038/nature03571. 15889048

[5] Lyles DS. 2000. Cytopathogenesis and inhibition of host gene expression by RNA viruses. Microbiol Mol Biol Rev 64:709–724. doi:10.1128/MMBR.64.4.709-724.2000. 11104816 PMC99011

[6] Schoggins JW. 2019. Interferon-stimulated genes: what do they all do? Annu Rev Virol 6:567–584. doi:10.1146/annurev-virology-092818-015756. 31283436

[7] Hyrcza MD, Kovacs C, Loutfy M, Halpenny R, Heisler L, Yang S, Wilkins O, Ostrowski M, Der SD. 2007. Distinct transcriptional profiles in ex vivo CD4^+^ and CD8^+^ T cells are established early in human immunodeficiency virus type 1 infection and are characterized by a chronic interferon response as well as extensive transcriptional changes in CD8^+^ T cells. J Virol 81:3477–3486. doi:10.1128/JVI.01552-06. 17251300 PMC1866039

[8] Sankaran S, Guadalupe M, Reay E, George MD, Flamm J, Prindiville T, Dandekar S. 2005. Gut mucosal T cell responses and gene expression correlate with protection against disease in long-term HIV-1-infected nonprogressors. Proc Natl Acad Sci U S A 102:9860–9865. doi:10.1073/pnas.0503463102. 15980151 PMC1159164

[9] Sedaghat AR, German J, Teslovich TM, Cofrancesco J, Jr, Jie CC, Talbot CC, Jr, Siliciano RF. 2008. Chronic CD4^+^ T-cell activation and depletion in human immunodeficiency virus type 1 infection: type I interferon-mediated disruption of T-cell dynamics. J Virol 82:1870–1883. doi:10.1128/JVI.02228-07. 18077723 PMC2258719

[10] Ivanov S, Lagunin A, Filimonov D, Tarasova O. 2020. Network-based analysis of OMICs data to understand the HIV-host interaction. Front Microbiol 11:1314. doi:10.3389/fmicb.2020.01314. 32625189 PMC7311653

[11] Giri MS, Nebozhyn M, Showe L, Montaner LJ. 2006. Microarray data on gene modulation by HIV-1 in immune cells: 2000–2006. J Leukoc Biol 80:1031–1043. doi:10.1189/jlb.0306157. 16940334

[12] Imbeault M, Giguere K, Ouellet M, Tremblay MJ. 2012. Exon level transcriptomic profiling of HIV-1-infected CD4(+) T cells reveals virus-induced genes and host environment favorable for viral replication. PLoS Pathog 8:e1002861. doi:10.1371/journal.ppat.1002861. 22876188 PMC3410884

[13] Sherrill-Mix S, Ocwieja KE, Bushman FD. 2015. Gene activity in primary T cells infected with HIV89.6: intron retention and induction of genomic repeats. Retrovirology 12:79. doi:10.1186/s12977-015-0205-1. 26377088 PMC4574318

[14] Langer S, Hammer C, Hopfensperger K, Klein L, Hotter D, De Jesus PD, Herbert KM, Pache L, Smith N, van der Merwe JA, Chanda SK, Fellay J, Kirchhoff F, Sauter D. 2019. HIV-1 Vpu is a potent transcriptional suppressor of NF-kappaB-elicited antiviral immune responses. Elife 8:e41930. doi:10.7554/eLife.41930. 30717826 PMC6372280

[15] Mohammadi P, Desfarges S, Bartha I, Joos B, Zangger N, Munoz M, Gunthard HF, Beerenwinkel N, Telenti A, Ciuffi A. 2013. 24 hours in the life of HIV-1 in a T cell line. PLoS Pathog 9:e1003161. doi:10.1371/journal.ppat.1003161. 23382686 PMC3561177

[16] Cicala C, Arthos J, Martinelli E, Censoplano N, Cruz CC, Chung E, Selig SM, Van Ryk D, Yang J, Jagannatha S, Chun TW, Ren P, Lempicki RA, Fauci AS. 2006. R5 and X4 HIV envelopes induce distinct gene expression profiles in primary peripheral blood mononuclear cells. Proc Natl Acad Sci U S A 103:3746–3751. doi:10.1073/pnas.0511237103. 16505369 PMC1533779

[17] Cicala C, Arthos J, Selig SM, Dennis G, Jr, Hosack DA, Van Ryk D, Spangler ML, Steenbeke TD, Khazanie P, Gupta N, Yang J, Daucher M, Lempicki RA, Fauci AS. 2002. HIV envelope induces a cascade of cell signals in non-proliferating target cells that favor virus replication. Proc Natl Acad Sci U S A 99:9380–9385. doi:10.1073/pnas.142287999. 12089333 PMC123149

[18] Rotger M, Dang KK, Fellay J, Heinzen EL, Feng S, Descombes P, Shianna KV, Ge DL, Gunthard HF, Goldstein DB, Telenti A, AID SHCSCH. 2010. Genome-wide mRNA expression correlates of viral control in CD4+ T-cells from HIV-1-infected individuals. PLoS Pathog 6:e1000781. doi:10.1371/journal.ppat.1000781. 20195503 PMC2829051

[19] Kleinman CL, Doria M, Orecchini E, Giuliani E, Galardi S, De Jay N, Michienzi A. 2014. HIV-1 infection causes a down-regulation of genes involved in ribosome biogenesis. PLoS One 9:e113908. doi:10.1371/journal.pone.0113908. 25462981 PMC4252078

[20] van 't Wout AB, Swain JV, Schindler M, Rao U, Pathmajeyan MS, Mullins JI, Kirchhoff F. 2005. Nef induces multiple genes involved in cholesterol synthesis and uptake in human immunodeficiency virus type 1-infected T cells. J Virol 79:10053–10058. doi:10.1128/JVI.79.15.10053-10058.2005. 16014965 PMC1181597

[21] Keele BF, Giorgi EE, Salazar-Gonzalez JF, Decker JM, Pham KT, Salazar MG, Sun C, Grayson T, Wang S, Li H, Wei X, Jiang C, Kirchherr JL, Gao F, Anderson JA, Ping LH, Swanstrom R, Tomaras GD, Blattner WA, Goepfert PA, Kilby JM, Saag MS, Delwart EL, Busch MP, Cohen MS, Montefiori DC, Haynes BF, Gaschen B, Athreya GS, Lee HY, Wood N, Seoighe C, Perelson AS, Bhattacharya T, Korber BT, Hahn BH, Shaw GM. 2008. Identification and characterization of transmitted and early founder virus envelopes in primary HIV-1 infection. Proc Natl Acad Sci U S A 105:7552–7557. doi:10.1073/pnas.0802203105. 18490657 PMC2387184

[22] Brenchley JM, Hill BJ, Ambrozak DR, Price DA, Guenaga FJ, Casazza JP, Kuruppu J, Yazdani J, Migueles SA, Connors M, Roederer M, Douek DC, Koup RA. 2004. T-cell subsets that harbor human immunodeficiency virus (HIV) in vivo: implications for HIV pathogenesis. J Virol 78:1160–1168. doi:10.1128/jvi.78.3.1160-1168.2004. 14722271 PMC321406

[23] Gao D, Wu J, Wu YT, Du F, Aroh C, Yan N, Sun L, Chen ZJ. 2013. Cyclic GMP-AMP synthase is an innate immune sensor of HIV and other retroviruses. Science 341:903–906. doi:10.1126/science.1240933. 23929945 PMC3860819

[24] Lepelley A, Louis S, Sourisseau M, Law HK, Pothlichet J, Schilte C, Chaperot L, Plumas J, Randall RE, Si-Tahar M, Mammano F, Albert ML, Schwartz O. 2011. Innate sensing of HIV-infected cells. PLoS Pathog 7:e1001284. doi:10.1371/journal.ppat.1001284. 21379343 PMC3040675

[25] Monroe KM, Yang Z, Johnson JR, Geng X, Doitsh G, Krogan NJ, Greene WC. 2014. IFI16 DNA sensor is required for death of lymphoid CD4 T cells abortively infected with HIV. Science 343:428–432. doi:10.1126/science.1243640. 24356113 PMC3976200

[26] Fletcher AJ, Vaysburd M, Maslen S, Zeng J, Skehel JM, Towers GJ, James LC. 2018. Trivalent RING assembly on retroviral capsids activates TRIM5 ubiquitination and innate immune signaling. Cell Host Microbe 24:761–775.e6. doi:10.1016/j.chom.2018.10.007. 30503508 PMC6299210

[27] Pertel T, Hausmann S, Morger D, Zuger S, Guerra J, Lascano J, Reinhard C, Santoni FA, Uchil PD, Chatel L, Bisiaux A, Albert ML, Strambio-De-Castillia C, Mothes W, Pizzato M, Grutter MG, Luban J. 2011. TRIM5 is an innate immune sensor for the retrovirus capsid lattice. Nature 472:361–365. doi:10.1038/nature09976. 21512573 PMC3081621

[28] Yoh SM, Schneider M, Seifried J, Soonthornvacharin S, Akleh RE, Olivieri KC, De Jesus PD, Ruan C, de Castro E, Ruiz PA, Germanaud D, Des Portes V, García-Sastre A, König R, Chanda SK. 2015. PQBP1 is a proximal sensor of the cGAS-dependent innate response to HIV-1. Cell 161:1293–1305. doi:10.1016/j.cell.2015.04.050. 26046437 PMC4503237

[29] Aldovini A, Young RA. 1990. Mutations of RNA and protein sequences involved in human immunodeficiency virus type 1 packaging result in production of noninfectious virus. J Virol 64:1920–1926. doi:10.1128/JVI.64.5.1920-1926.1990. 2109098 PMC249345

[30] Ding P, Kharytonchyk S, Waller A, Mbaekwe U, Basappa S, Kuo N, Frank HM, Quasney C, Kidane A, Swanson C, Van V, Sarkar M, Cannistraci E, Chaudhary R, Flores H, Telesnitsky A, Summers MF. 2020. Identification of the initial nucleocapsid recognition element in the HIV-1 RNA packaging signal. Proc Natl Acad Sci U S A 117:17737–17746. doi:10.1073/pnas.2008519117. 32647061 PMC7395439

[31] Fisher AG, Feinberg MB, Josephs SF, Harper ME, Marselle LM, Reyes G, Gonda MA, Aldovini A, Debouk C, Gallo RC, Wong-Staal F. 1986. The trans-activator gene of HTLV-III is essential for virus replication. Nature 320:367–371. doi:10.1038/320367a0. 3007995

[32] Jenkins Y, Pornillos O, Rich RL, Myszka DG, Sundquist WI, Malim MH. 2001. Biochemical analyses of the interactions between human immunodeficiency virus type 1 Vpr and p6^Gag^. J Virol 75:10537–10542. doi:10.1128/JVI.75.21.10537-10542.2001. 11581428 PMC114634

[33] Cohen EA, Dehni G, Sodroski JG, Haseltine WA. 1990. Human immunodeficiency virus vpr product is a virion-associated regulatory protein. J Virol 64:3097–3099. doi:10.1128/JVI.64.6.3097-3099.1990. 2139896 PMC249501

[34] Paxton W, Connor RI, Landau NR. 1993. Incorporation of Vpr into human immunodeficiency virus type 1 virions: requirement for the p6 region of gag and mutational analysis. J Virol 67:7229–7237. doi:10.1128/JVI.67.12.7229-7237.1993. 8230445 PMC238185

[35] Guenzel CA, Hérate C, Benichou S. 2014. HIV-1 Vpr-a still “enigmatic multitasker.” Front Microbiol 5:127. doi:10.3389/fmicb.2014.00127. 24744753 PMC3978352

[36] Fabryova H, Strebel K. 2019. Vpr and its cellular interaction partners: R we there yet? Cells 8:1310. doi:10.3390/cells8111310. PMC6912716 31652959

[37] Wallet C, Rohr O, Schwartz C. 2020. Evolution of a concept: from accessory protein to key virulence factor, the case of HIV-1 Vpr. Biochem Pharmacol 180:114128. doi:10.1016/j.bcp.2020.114128. 32619426

[38] Doehle BP, Hladik F, McNevin JP, McElrath MJ, Gale M. Jr, 2009. Human immunodeficiency virus type 1 mediates global disruption of innate antiviral signaling and immune defenses within infected cells. J Virol 83:10395–10405. doi:10.1128/JVI.00849-09. 19706707 PMC2753137

[39] Okumura A, Alce T, Lubyova B, Ezelle H, Strebel K, Pitha PM. 2008. HIV-1 accessory proteins VPR and Vif modulate antiviral response by targeting IRF-3 for degradation. Virology 373:85–97. doi:10.1016/j.virol.2007.10.042. 18082865 PMC2312338

[40] Trotard M, Tsopoulidis N, Tibroni N, Willemsen J, Binder M, Ruggieri A, Fackler OT. 2016. Sensing of HIV-1 infection in Tzm-bl cells with reconstituted expression of STING. J Virol 90:2064–2076. doi:10.1128/JVI.02966-15. 26656698 PMC4733976

[41] Vermeire J, Roesch F, Sauter D, Rua R, Hotter D, Van Nuffel A, Vanderstraeten H, Naessens E, Iannucci V, Landi A, Witkowski W, Baeyens A, Kirchhoff F, Verhasselt B. 2016. HIV triggers a cGAS-dependent, Vpu- and Vpr-regulated type I interferon response in CD4(+) T cells. Cell Rep 17:413–424. doi:10.1016/j.celrep.2016.09.023. 27705790

[42] Chang ST, Sova P, Peng X, Weiss J, Law GL, Palermo RE, Katze MG. 2011. Next-generation sequencing reveals HIV-1-mediated suppression of T cell activation and RNA processing and regulation of noncoding RNA expression in a CD4^+^ T cell line. mBio 2:e00134-11. doi:10.1128/mBio.00134-11. 21933919 PMC3175625

[43] Lefebvre G, Desfarges S, Uyttebroeck F, Munoz M, Beerenwinkel N, Rougemont J, Telenti A, Ciuffi A. 2011. Analysis of HIV-1 expression level and sense of transcription by high-throughput sequencing of the infected cell. J Virol 85:6205–6211. doi:10.1128/JVI.00252-11. 21507965 PMC3126515

[44] Jowett JB, Planelles V, Poon B, Shah NP, Chen ML, Chen IS. 1995. The human immunodeficiency virus type 1 vpr gene arrests infected T cells in the G_2_ + M phase of the cell cycle. J Virol 69:6304–6313. doi:10.1128/JVI.69.10.6304-6313.1995. 7666531 PMC189529

[45] Rogel ME, Wu LI, Emerman M. 1995. The human immunodeficiency virus type 1 vpr gene prevents cell proliferation during chronic infection. J Virol 69:882–888. doi:10.1128/JVI.69.2.882-888.1995. 7815556 PMC188655

[46] Re F, Braaten D, Franke EK, Luban J. 1995. Human immunodeficiency virus type 1 Vpr arrests the cell cycle in G2 by inhibiting the activation of p34cdc2-cyclin B. J Virol 69:6859–6864. doi:10.1128/jvi.69.11.6859-6864.1995. 7474100 PMC189600

[47] He J, Choe S, Walker R, Di Marzio P, Morgan DO, Landau NR. 1995. Human immunodeficiency virus type 1 viral protein R (Vpr) arrests cells in the G_2_ phase of the cell cycle by inhibiting p34cdc2 activity. J Virol 69:6705–6711. doi:10.1128/JVI.69.11.6705-6711.1995. 7474080 PMC189580

[48] Roux P, Alfieri C, Hrimech M, Cohen EA, Tanner JE. 2000. Activation of transcription factors NF-kappaB and NF-IL-6 by human immunodeficiency virus type 1 protein R (Vpr) induces interleukin-8 expression. J Virol 74:4658–4665. doi:10.1128/jvi.74.10.4658-4665.2000. 10775602 PMC111986

[49] Liu R, Lin Y, Jia R, Geng Y, Liang C, Tan J, Qiao W. 2014. HIV-1 Vpr stimulates NF-kappaB and AP-1 signaling by activating TAK1. Retrovirology 11:45. doi:10.1186/1742-4690-11-45. 24912525 PMC4057933

[50] Muthumani K, Choo AY, Zong WX, Madesh M, Hwang DS, Premkumar A, Thieu KP, Emmanuel J, Kumar S, Thompson CB, Weiner DB. 2006. The HIV-1 Vpr and glucocorticoid receptor complex is a gain-of-function interaction that prevents the nuclear localization of PARP-1. Nat Cell Biol 8:170–179. doi:10.1038/ncb1352. 16429131 PMC3142937

[51] Kogan M, Deshmane S, Sawaya BE, Gracely EJ, Khalili K, Rappaport J. 2013. Inhibition of NF-kappaB activity by HIV-1 Vpr is dependent on Vpr binding protein. J Cell Physiol 228:781–790. doi:10.1002/jcp.24226. 23001849 PMC3604695

[52] Khan H, Sumner RP, Rasaiyaah J, Tan CP, Rodriguez-Plata MT, Van Tulleken C, Fink D, Zuliani-Alvarez L, Thorne L, Stirling D, Milne RS, Towers GJ. 2020. HIV-1 Vpr antagonizes innate immune activation by targeting karyopherin-mediated NF-κB/IRF3 nuclear transport. Elife 9:e60821. doi:10.7554/eLife.60821. 33300875 PMC7759385

[53] Zahoor MA, Xue G, Sato H, Murakami T, Takeshima SN, Aida Y. 2014. HIV-1 Vpr induces interferon-stimulated genes in human monocyte-derived macrophages. PLoS One 9:e106418. doi:10.1371/journal.pone.0106418. 25170834 PMC4149569

[54] Rasaiyaah J, Tan CP, Fletcher AJ, Price AJ, Blondeau C, Hilditch L, Jacques DA, Selwood DL, James LC, Noursadeghi M, Towers GJ. 2013. HIV-1 evades innate immune recognition through specific cofactor recruitment. Nature 503:402–405. doi:10.1038/nature12769. 24196705 PMC3928559

[55] Miller CM, Akiyama H, Agosto LM, Emery A, Ettinger CR, Swanstrom RI, Henderson AJ, Gummuluru S. 2017. Virion-associated Vpr alleviates a postintegration block to HIV-1 infection of dendritic cells. J Virol 91:e00051-17. doi:10.1128/JVI.00051-17. 28424288 PMC5469257

[56] Dupont L, Bloor S, Williamson JC, Cuesta SM, Shah R, Teixeira-Silva A, Naamati A, Greenwood EJD, Sarafianos SG, Matheson NJ, Lehner PJ. 2021. The SMC5/6 complex compacts and silences unintegrated HIV-1 DNA and is antagonized by Vpr. Cell Host Microbe 29:792–805.e6. doi:10.1016/j.chom.2021.03.001. 33811831 PMC8118623

[57] Belzile JP, Duisit G, Rougeau N, Mercier J, Finzi A, Cohen EA. 2007. HIV-1 Vpr-mediated G2 arrest involves the DDB1-CUL4AVPRBP E3 ubiquitin ligase. PLoS Pathog 3:e85. doi:10.1371/journal.ppat.0030085. 17630831 PMC1914068

[58] Hrecka K, Gierszewska M, Srivastava S, Kozaczkiewicz L, Swanson SK, Florens L, Washburn MP, Skowronski J. 2007. Lentiviral Vpr usurps Cul4-DDB1[VprBP] E3 ubiquitin ligase to modulate cell cycle. Proc Natl Acad Sci U S A 104:11778–11783. doi:10.1073/pnas.0702102104. 17609381 PMC1906728

[59] Le Rouzic E, Belaidouni N, Estrabaud E, Morel M, Rain JC, Transy C, Margottin-Goguet F. 2007. HIV1 Vpr arrests the cell cycle by recruiting DCAF1/VprBP, a receptor of the Cul4-DDB1 ubiquitin ligase. Cell Cycle 6:182–188. doi:10.4161/cc.6.2.3732. 17314515

[60] Schrofelbauer B, Hakata Y, Landau NR. 2007. HIV-1 Vpr function is mediated by interaction with the damage-specific DNA-binding protein DDB1. Proc Natl Acad Sci U S A 104:4130–4135. doi:10.1073/pnas.0610167104. 17360488 PMC1820720

[61] Tan L, Ehrlich E, Yu XF. 2007. DDB1 and Cul4A are required for human immunodeficiency virus type 1 Vpr-induced G_2_ arrest. J Virol 81:10822–10830. doi:10.1128/JVI.01380-07. 17626091 PMC2045451

[62] Wen X, Duus KM, Friedrich TD, de Noronha CM. 2007. The HIV1 protein Vpr acts to promote G2 cell cycle arrest by engaging a DDB1 and Cullin4A-containing ubiquitin ligase complex using VprBP/DCAF1 as an adaptor. J Biol Chem 282:27046–27057. doi:10.1074/jbc.M703955200. 17620334

[63] Yan J, Shun MC, Hao C, Zhang Y, Qian J, Hrecka K, DeLucia M, Monnie C, Ahn J, Skowronski J. 2018. HIV-1 Vpr reprograms CLR4(DCAF1) E3 ubiquitin ligase to antagonize exonuclease 1-mediated restriction of HIV-1 infection. mBio 9:e01732-18. doi:10.1128/mBio.01732-18. 30352932 PMC6199497

[64] Wu Y, Zhou X, Barnes CO, DeLucia M, Cohen AE, Gronenborn AM, Ahn J, Calero G. 2016. The DDB1-DCAF1-Vpr-UNG2 crystal structure reveals how HIV-1 Vpr steers human UNG2 toward destruction. Nat Struct Mol Biol 23:933–940. doi:10.1038/nsmb.3284. 27571178 PMC5385928

[65] Greenwood EJD, Williamson JC, Sienkiewicz A, Naamati A, Matheson NJ, Lehner PJ. 2019. Promiscuous targeting of cellular proteins by Vpr drives systems-level proteomic remodeling in HIV-1 infection. Cell Rep 27:1579–1596.e7. doi:10.1016/j.celrep.2019.04.025. 31042482 PMC6506760

[66] Romani B, Baygloo NS, Hamidi-Fard M, Aghasadeghi MR, Allahbakhshi E. 2016. HIV-1 Vpr protein induces proteasomal degradation of chromatin-associated class I HDACs to overcome latent infection of macrophages. J Biol Chem 291:2696–2711. doi:10.1074/jbc.M115.689018. 26679995 PMC4742738

[67] Romani B, Kamali Jamil R, Hamidi-Fard M, Rahimi P, Momen SB, Aghasadeghi MR, Allahbakhshi E. 2016. HIV-1 Vpr reactivates latent HIV-1 provirus by inducing depletion of class I HDACs on chromatin. Sci Rep 6:31924. doi:10.1038/srep31924. 27550312 PMC4994036

[68] Maudet C, Sourisce A, Dragin L, Lahouassa H, Rain JC, Bouaziz S, Ramirez BC, Margottin-Goguet F. 2013. HIV-1 Vpr induces the degradation of ZIP and sZIP, adaptors of the NuRD chromatin remodeling complex, by hijacking DCAF1/VprBP. PLoS One 8:e77320. doi:10.1371/journal.pone.0077320. 24116224 PMC3792905

[69] Lv L, Wang Q, Xu Y, Tsao L-C, Nakagawa T, Guo H, Su L, Xiong Y. 2018. Vpr targets TET2 for degradation by CRL4(VprBP) E3 ligase to sustain IL-6 expression and enhance HIV-1 replication. Mol Cell 70:961–970.e5. doi:10.1016/j.molcel.2018.05.007. 29883611 PMC6071318

[70] Chougui G, Munir-Matloob S, Matkovic R, Martin MM, Morel M, Lahouassa H, Leduc M, Ramirez BC, Etienne L, Margottin-Goguet F. 2018. HIV-2/SIV viral protein X counteracts HUSH repressor complex. Nat Microbiol 3:891–897. doi:10.1038/s41564-018-0179-6. 29891865

[71] Yurkovetskiy L, Guney MH, Kim K, Goh SL, McCauley S, Dauphin A, Diehl WE, Luban J. 2018. Primate immunodeficiency virus proteins Vpx and Vpr counteract transcriptional repression of proviruses by the HUSH complex. Nat Microbiol 3:1354–1361. doi:10.1038/s41564-018-0256-x. 30297740 PMC6258279

[72] Levy DN, Aldrovandi GM, Kutsch O, Shaw GM. 2004. Dynamics of HIV-1 recombination in its natural target cells. Proc Natl Acad Sci U S A 101:4204–4209. doi:10.1073/pnas.0306764101. 15010526 PMC384719

[73] Weiss SH, Goedert JJ, Gartner S, Popovic M, Waters D, Markham P, di Marzo Veronese F, Gail MH, Barkley WE, Gibbons J. 1988. Risk of human immunodeficiency virus (HIV-1) infection among laboratory workers. Science 239:68–71. doi:10.1126/science.3336776. 3336776

[74] Beaumont T, van Nuenen A, Broersen S, Blattner WA, Lukashov VV, Schuitemaker H. 2001. Reversal of human immunodeficiency virus type 1 IIIB to a neutralization-resistant phenotype in an accidentally infected laboratory worker with a progressive clinical course. J Virol 75:2246–2252. doi:10.1128/JVI.75.5.2246-2252.2001. 11160728 PMC114808

[75] Lang SM, Weeger M, Stahl-Hennig C, Coulibaly C, Hunsmann G, Muller J, Muller-Hermelink H, Fuchs D, Wachter H, Daniel MM. 1993. Importance of vpr for infection of rhesus monkeys with simian immunodeficiency virus. J Virol 67:902–912. doi:10.1128/JVI.67.2.902-912.1993. 8380472 PMC237444

[76] Gibbs JS, Lackner AA, Lang SM, Simon MA, Sehgal PK, Daniel MD, Desrosiers RC. 1995. Progression to AIDS in the absence of a gene for vpr or vpx. J Virol 69:2378–2383. doi:10.1128/JVI.69.4.2378-2383.1995. 7884883 PMC188910

[77] Goh WC, Rogel ME, Kinsey CM, Michael SF, Fultz PN, Nowak MA, Hahn BH, Emerman M. 1998. HIV-1 Vpr increases viral expression by manipulation of the cell cycle: a mechanism for selection of Vpr in vivo. Nat Med 4:65–71. doi:10.1038/nm0198-065. 9427608

[78] Tzitzivacos DB, Tiemessen CT, Stevens WS, Papathanasopoulos MA. 2009. Viral genetic determinants of nonprogressive HIV type 1 subtype C infection in antiretroviral drug-naive children. AIDS Res Hum Retroviruses 25:1141–1148. doi:10.1089/aid.2009.0080. 19895210

[79] Ali A, Ng HL, Blankson JN, Burton DR, Buckheit RW, III, Moldt B, Fulcher JA, Ibarrondo FJ, Anton PA, Yang OO. 2018. Highly attenuated infection with a Vpr-deleted molecular clone of human immunodeficiency virus-1. J Infect Dis 218:1447–1452. doi:10.1093/infdis/jiy346. 29878133 PMC6151090

[80] Simon JH, Southerling TE, Peterson JC, Meyer BE, Malim MH. 1995. Complementation of vif-defective human immunodeficiency virus type 1 by primate, but not nonprimate, lentivirus vif genes. J Virol 69:4166–4172. doi:10.1128/JVI.69.7.4166-4172.1995. 7769676 PMC189153

[81] Garrett ED, Tiley LS, Cullen BR. 1991. Rev activates expression of the human immunodeficiency virus type 1 vif and vpr gene products. J Virol 65:1653–1657. doi:10.1128/JVI.65.3.1653-1657.1991. 1825343 PMC239957

[82] Simon JH, Gaddis NC, Fouchier RA, Malim MH. 1998. Evidence for a newly discovered cellular anti-HIV-1 phenotype. Nat Med 4:1397–1400. doi:10.1038/3987. 9846577

[83] Ochsenbauer C, Edmonds TG, Ding H, Keele BF, Decker J, Salazar MG, Salazar-Gonzalez JF, Shattock R, Haynes BF, Shaw GM, Hahn BH, Kappes JC. 2012. Generation of transmitted/founder HIV-1 infectious molecular clones and characterization of their replication capacity in CD4 T lymphocytes and monocyte-derived macrophages. J Virol 86:2715–2728. doi:10.1128/JVI.06157-11. 22190722 PMC3302286

[84] Gartner S, Markovits P, Markovitz DM, Kaplan MH, Gallo RC, Popovic M. 1986. The role of mononuclear phagocytes in HTLV-III/LAV infection. Science 233:215–219. doi:10.1126/science.3014648. 3014648

[85] Jagodzinski LL, Wiggins DL, McManis JL, Emery S, Overbaugh J, Robb M, Bodrug S, Michael NL. 2000. Use of calibrated viral load standards for group M subtypes of human immunodeficiency virus type 1 to assess the performance of viral RNA quantitation tests. J Clin Microbiol 38:1247–1249. doi:10.1128/JCM.38.3.1247-1249.2000. 10699033 PMC86389

[86] Malim MH, Hauber J, Fenrick R, Cullen BR. 1988. Immunodeficiency virus rev trans-activator modulates the expression of the viral regulatory genes. Nature 335:181–183. doi:10.1038/335181a0. 3412474

[87] Gaddis NC, Sheehy AM, Ahmad KM, Swanson CM, Bishop KN, Beer BE, Marx PA, Gao F, Bibollet-Ruche F, Hahn BH, Malim MH. 2004. Further investigation of simian immunodeficiency virus Vif function in human cells. J Virol 78:12041–12046. doi:10.1128/JVI.78.21.12041-12046.2004. 15479843 PMC523299

[88] Simon JH, Fouchier RA, Southerling TE, Guerra CB, Grant CK, Malim MH. 1997. The Vif and Gag proteins of human immunodeficiency virus type 1 colocalize in infected human T cells. J Virol 71:5259–5267. doi:10.1128/JVI.71.7.5259-5267.1997. 9188594 PMC191762

[89] O'Doherty U, Swiggard WJ, Malim MH. 2000. Human immunodeficiency virus type 1 spinoculation enhances infection through virus binding. J Virol 74:10074–10080. doi:10.1128/jvi.74.21.10074-10080.2000. 11024136 PMC102046

[90] Goujon C, Malim MH. 2010. Characterization of the alpha interferon-induced postentry block to HIV-1 infection in primary human macrophages and T cells. J Virol 84:9254–9266. doi:10.1128/JVI.00854-10. 20610724 PMC2937661

[91] Smyth GK. 2004. Linear models and empirical bayes methods for assessing differential expression in microarray experiments. Stat Appl Genet Mol Biol 3:Article3. doi:10.2202/1544-6115.1027. 16646809

[92] Shi W, Oshlack A, Smyth GK. 2010. Optimizing the noise versus bias trade-off for Illumina whole genome expression BeadChips. Nucleic Acids Res 38:e204. doi:10.1093/nar/gkq871. 20929874 PMC3001098

[93] McCarthy DJ, Smyth GK. 2009. Testing significance relative to a fold-change threshold is a TREAT. Bioinformatics 25:765–771. doi:10.1093/bioinformatics/btp053. 19176553 PMC2654802

[94] Ritchie ME, Diyagama D, Neilson J, van Laar R, Dobrovic A, Holloway A, Smyth GK. 2006. Empirical array quality weights in the analysis of microarray data. BMC Bioinformatics 7:261. doi:10.1186/1471-2105-7-261. 16712727 PMC1564422

[95] Lex A, Gehlenborg N, Strobelt H, Vuillemot R, Pfister H. 2014. UpSet: visualization of intersecting sets. IEEE Trans Vis Comput Graph 20:1983–1992. doi:10.1109/TVCG.2014.2346248. 26356912 PMC4720993

[96] Langfelder P, Zhang B, Horvath S. 2008. Defining clusters from a hierarchical cluster tree: the Dynamic Tree Cut package for R. Bioinformatics 24:719–720. doi:10.1093/bioinformatics/btm563. 18024473

[97] Leek JT, Johnson WE, Parker HS, Jaffe AE, Storey JD. 2012. The sva package for removing batch effects and other unwanted variation in high-throughput experiments. Bioinformatics 28:882–883. doi:10.1093/bioinformatics/bts034. 22257669 PMC3307112

[98] Reimand J, Kull M, Peterson H, Hansen J, Vilo J. 2007. g:Profiler—a web-based toolset for functional profiling of gene lists from large-scale experiments. Nucleic Acids Res 35:W193–W200. doi:10.1093/nar/gkm226. 17478515 PMC1933153

[99] Martin M. 2011. Cutadapt removes adapter sequences from high-throughput sequencing reads. EMBnetjournal 17:10–12. doi:10.14806/ej.17.1.200.

[100] Kim D, Langmead B, Salzberg SL. 2015. HISAT: a fast spliced aligner with low memory requirements. Nat Methods 12:357–360. doi:10.1038/nmeth.3317. 25751142 PMC4655817

[101] Lawrence M, Huber W, Pagès H, Aboyoun P, Carlson M, Gentleman R, Morgan MT, Carey VJ. 2013. Software for computing and annotating genomic ranges. PLoS Comput Biol 9:e1003118. doi:10.1371/journal.pcbi.1003118. 23950696 PMC3738458

[102] Love MI, Huber W, Anders S. 2014. Moderated estimation of fold change and dispersion for RNA-seq data with DESeq2. Genome Biol 15:550. doi:10.1186/s13059-014-0550-8. 25516281 PMC4302049

[103] Robinson MD, McCarthy DJ, Smyth GK. 2010. edgeR: a Bioconductor package for differential expression analysis of digital gene expression data. Bioinformatics 26:139–140. doi:10.1093/bioinformatics/btp616. 19910308 PMC2796818

[104] Ritchie ME, Phipson B, Wu D, Hu Y, Law CW, Shi W, Smyth GK. 2015. limma powers differential expression analyses for RNA-sequencing and microarray studies. Nucleic Acids Res 43:e47. doi:10.1093/nar/gkv007. 25605792 PMC4402510

