# Supplementary figures and images for: HIV-1 Vpr Induces Widespread Transcriptomic Changes in CD4+ T Cells Early Postinfection
Source: mBio. 2021 Jun 22;12(3):e01369-21. doi: 10.1128/mBio.01369-21 (PMC8263007; doi:10.1128/mBio.01369-21)

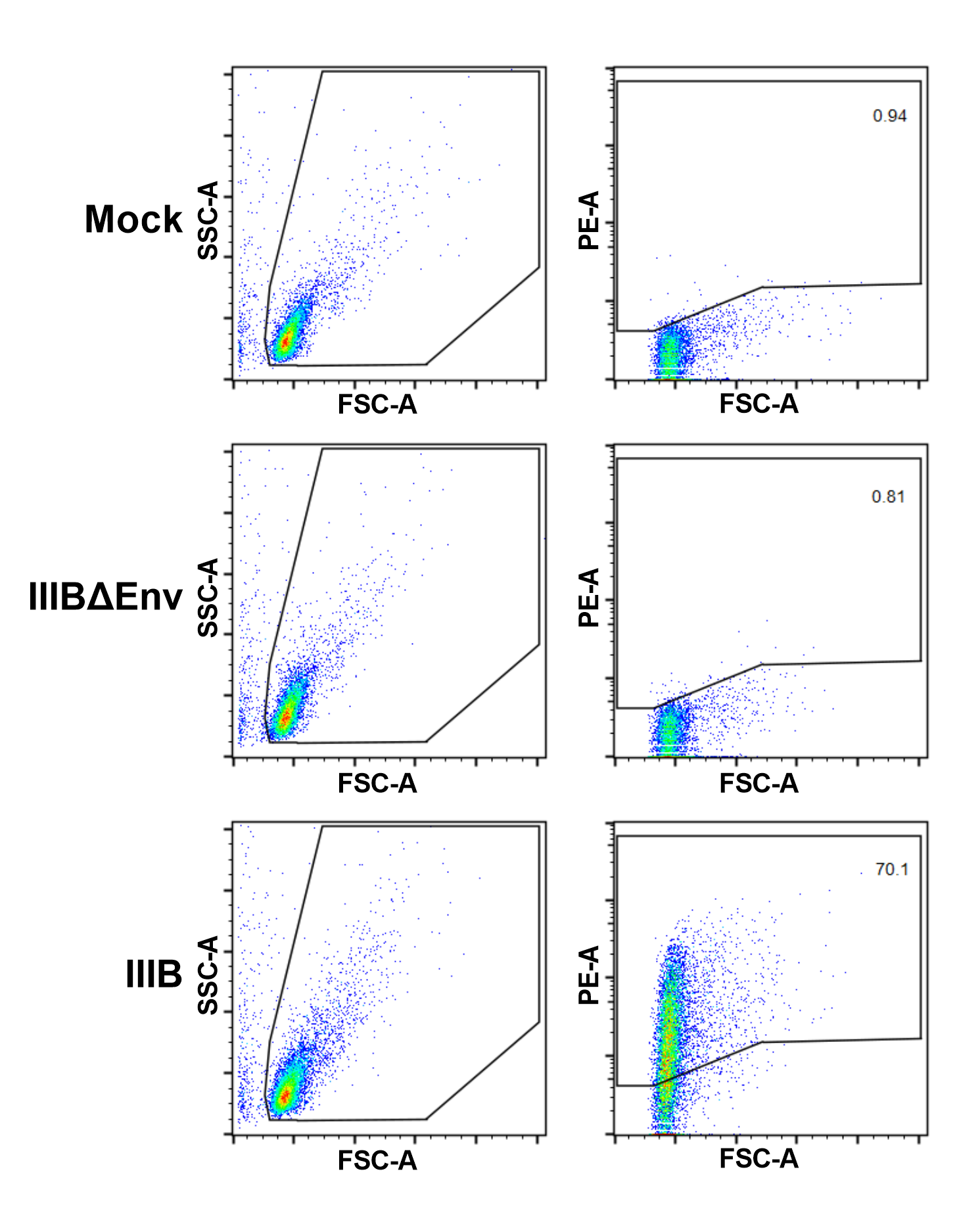

Supplement: FIG S2 [file mbio.01369-21-sf002.tif]

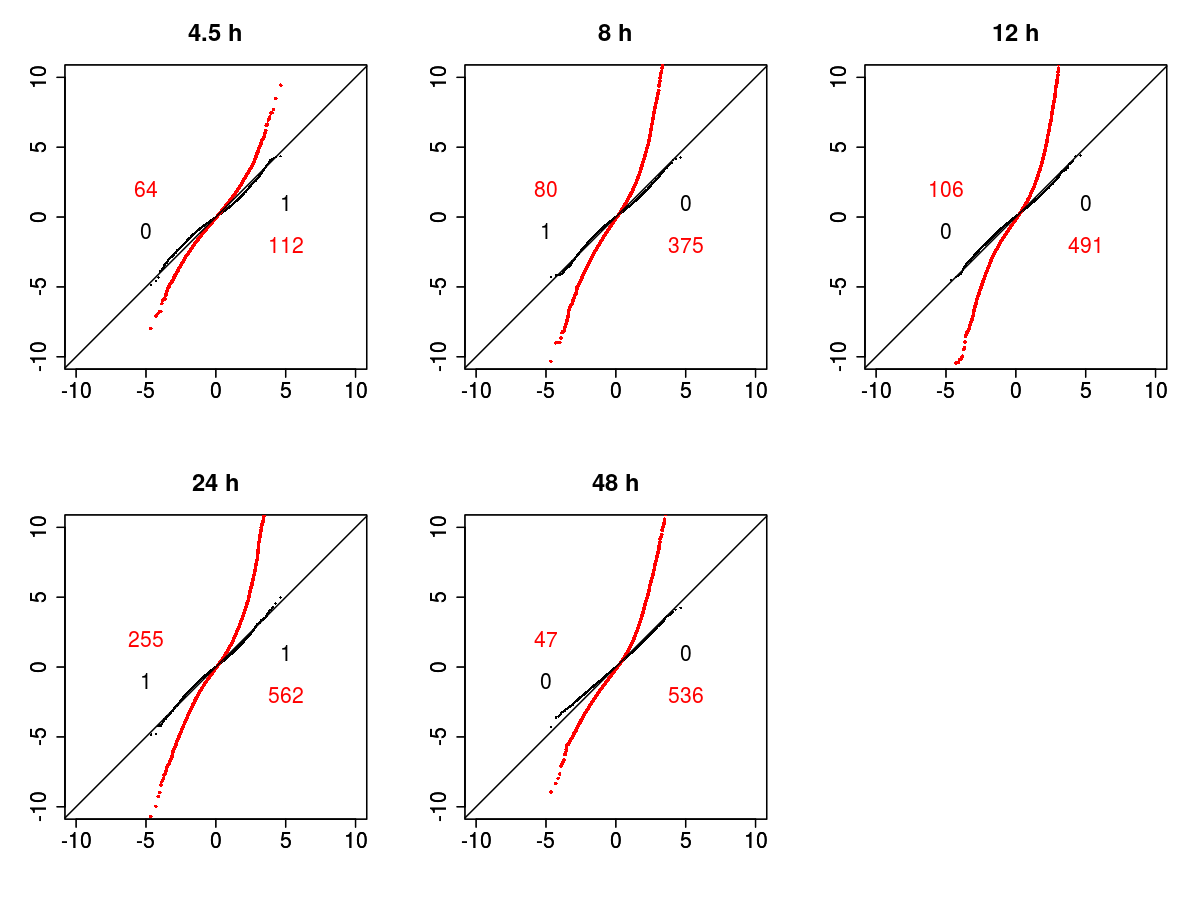

Supplement: FIG S3 [file mbio.01369-21-sf003.tif]

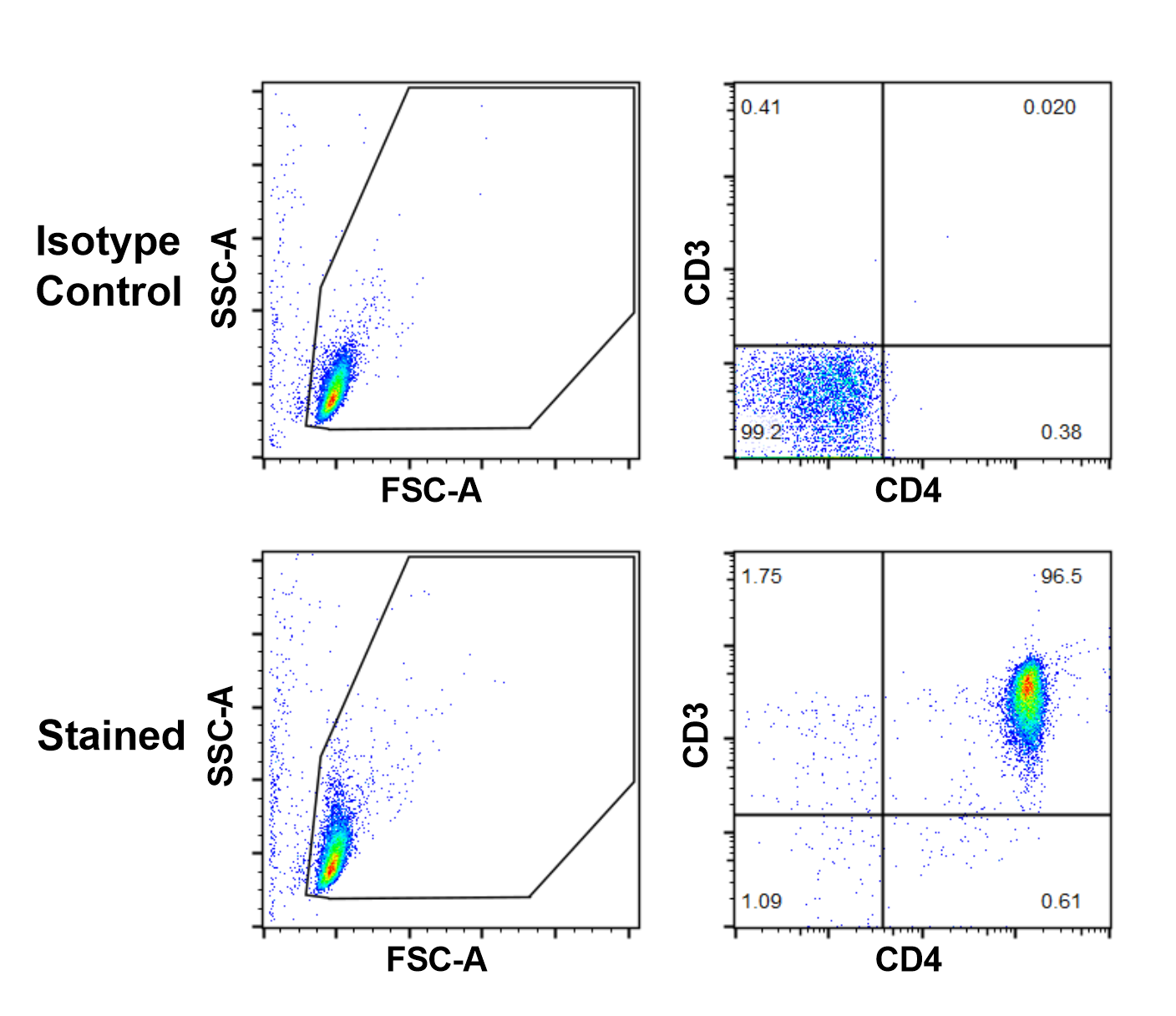

Supplement: FIG S1 [file mbio.01369-21-sf001.tif]
